# Anti-Diabetic Therapies and Cancer: From Bench to Bedside

**DOI:** 10.3390/biom14111479

**Published:** 2024-11-20

**Authors:** Dimitris Kounatidis, Natalia G. Vallianou, Irene Karampela, Eleni Rebelos, Marina Kouveletsou, Vasileios Dalopoulos, Petros Koufopoulos, Evanthia Diakoumopoulou, Nikolaos Tentolouris, Maria Dalamaga

**Affiliations:** 1 Diabetes Center, First Department of Propaedeutic Internal Medicine, Medical School, Laiko General Hospital, National and Kapustina University of Athens, 11527 Athens, Greece; dimitriskounatidis82@outlook.com (D.K.); eleni.rebelos@utu.fi (E.R.); marinakouveletsou@gmail.com (M.K.); evitadiak@gmail.com (E.D.); ntentol@med.uoa.gr (N.T.); 2 First Department of Internal Medicine, Sismanogleio General Hospital, 15126 Athens, Greece; natalia.vallianou@hotmail.com (N.G.V.); billydalo@hotmail.gr (V.D.); peterkouf13@gmail.com (P.K.); 3 2nd Department of Critical Care, Medical School, Attikon General University Hospital, University of Athens, 1 Rimini str., 12461 Athens, Greece; eikaras1@gmail.com; 4 Department of Biological Chemistry, National and Kapodistrian University of Athens, 75 Mikras Asias str., 11527 Athens, Greece

**Keywords:** cancer, chronic low-grade inflammation, diabetes mellitus, doxorubicin-induced cardiomyopathy, GLP-1 receptor agonists, immune check point inhibitors, metformin, SGLT-2 inhibitors, tirzepatide, tumor microenvironment

## Abstract

Diabetes mellitus (DM) is a significant risk factor for various cancers, with the impact of anti-diabetic therapies on cancer progression differing across malignancies. Among these therapies, metformin has gained attention for its potential anti-cancer effects, primarily through modulation of the AMP-activated protein kinase/mammalian target of rapamycin (AMPK/mTOR) pathway and the induction of autophagy. Beyond metformin, other conventional anti-diabetic treatments, such as insulin, sulfonylureas (SUs), pioglitazone, and dipeptidyl peptidase-4 (DPP-4) inhibitors, have also been examined for their roles in cancer biology, though findings are often inconclusive. More recently, novel medications, like glucagon-like peptide-1 (GLP-1) receptor agonists, dual GLP-1/glucose-dependent insulinotropic polypeptide (GIP) agonists, and sodium-glucose co-transporter-2 (SGLT-2) inhibitors, have revolutionized DM management by not only improving glycemic control but also delivering substantial cardiovascular and renal benefits. Given their diverse metabolic effects, including anti-obesogenic properties, these novel agents are now under meticulous investigation for their potential influence on tumorigenesis and cancer advancement. This review aims to offer a comprehensive exploration of the evolving landscape of glucose-lowering treatments and their implications in cancer biology. It critically evaluates experimental evidence surrounding the molecular mechanisms by which these medications may modulate oncogenic signaling pathways and reshape the tumor microenvironment (TME). Furthermore, it assesses translational research and clinical trials to gauge the practical relevance of these findings in real-world settings. Finally, it explores the potential of anti-diabetic medications as adjuncts in cancer treatment, particularly in enhancing the efficacy of chemotherapy, minimizing toxicity, and addressing resistance within the framework of immunotherapy.

## 1. Introduction

Diabetes mellitus (DM), affecting approximately 537 million adults globally and projected to increase to 643 million by 2030, represents a significant global health crisis [1]. Notably, DM has long been recognized for its intricate bidirectional relationship with malignancy, influencing cancer prognosis and outcomes [2]. Current evidence shows cancer as the second leading cause of mortality in type 1 diabetes mellitus (T1DM), with nearly 20% of cancer patients concurrently diagnosed with DM [3,4]. This relationship is believed to stem from shared pathophysiological mechanisms, including hyperinsulinemia, chronic low-grade inflammation, and oxidative stress, promoting a cellular environment conducive to tumorigenesis [5]. Within this framework, anti-diabetic drugs have garnered attention, with studies suggesting both protective and carcinogenic potentials across various agents [6]. While certain drugs are contraindicated by the World Health Organization (WHO) for specific cancer types, conflicting studies complicate these guidelines [7].

Among these therapies, metformin has gained attention for its potential anti-cancer properties, primarily attributed to the activation of the AMP-activated protein kinase (AMPK) pathway, alongside its role in glucose regulation [8]. Other conventional anti-diabetic drugs have also been scrutinized, with evidence linking hyperinsulinemia to cancer risk, raising concerns about insulin therapy’s carcinogenic potential, though the data remain ambiguous [9]. Additionally, pioglitazone, a peroxisome proliferator-activated receptor gamma (PPAR-γ) agonist, has been associated with an elevated bladder cancer risk, prompting caution in clinical guidelines, yet exhibits promising effects in cancers of the breast, lung, and colon [10].

The elucidation of the incretin effect has catalyzed the development of cutting-edge therapies that have markedly transformed the management of type 2 diabetes mellitus (T2DM). Mediated predominantly by the incretin hormones glucose-dependent insulinotropic polypeptide (GIP) and glucagon-like peptide-1 (GLP-1), this effect enhances insulin secretion in response to oral glucose intake, playing a vital role in maintaining glucose homeostasis. The enzyme dipeptidyl peptidase-4 (DPP-4) regulates the duration of incretin hormone activity, thereby influencing its metabolic effects [11]. Incretin-based therapies currently include DPP-4 inhibitors and GLP-1 receptor agonists (GLP-1 RAs), with the recent introduction of the dual GIP/GLP-1 agonist tirzepatide [12,13]. While GLP-1 RAs and tirzepatide have demonstrated essential cardiovascular (CV) benefits, concerns regarding their associations with thyroid and pancreatic cancers have emerged, leading to cautionary guidelines. Nonetheless, given their impact on weight reduction, these therapies may, under certain conditions, offer protective effects against malignancy [14,15].

Sodium-glucose co-transporter-2 (SGLT-2) inhibitors, a distinct class of anti-diabetic drugs, lower blood glucose levels by promoting glycosuria through the inhibition of the SGLT-2 co-transporter in the proximal renal tubules [16]. Noted for their versatility in T2DM management and benefits in heart failure and chronic kidney disease (CKD), SGLT-2 inhibitors were also explored for their anti-cancer qualities, with promising preclinical and clinical findings [17].

Through their diverse mechanisms of action, glucose-lowering therapies modulate key oncogenic signaling pathways and significantly alter the tumor microenvironment (TME). The TME plays a critical role in cancer biology, significantly influencing tumor initiation, progression, and metastasis. Comprised of a complex network of cellular and non-cellular elements, the TME fosters a niche that supports tumor growth and survival through mechanisms such as immune evasion, angiogenesis, and the release of pro-tumorigenic cytokines and growth factors. This network is shaped by interactions among immune cells, including T and B lymphocytes, natural killers (NK) cells, dendritic cells (DCs), and macrophages, as well as stromal components such as cancer-associated fibroblasts (CAFs), endothelial cells, and adipocytes. Additionally, extracellular matrix components and non-cellular factors like exosomes also contribute to the formation of a pro-tumorigenic TME [18]. However, despite the breadth of research, the overall impact of anti-diabetic medications on cancer risk and progression remains inconclusive, necessitating further investigation [7,19].

This narrative review explored the intersection between anti-diabetic treatments and cancer biology, focusing on how these treatments influence cancer-related signaling pathways and the TME. It further examined the translation of experimental findings into clinical practice, highlighting their potential to impact therapeutic outcomes, including improving chemotherapy efficacy, reducing toxicity, and overcoming resistance in immunotherapy.

## 2. Anti-Diabetic Therapies and Their Impact on Cancer Reprogramming: Insights from Experimental Studies

### 2.1. Metformin

Among the various classes of anti-DM medications, metformin stands out as the most extensively studied agent in relation to its effects on carcinogenesis. Over the years, substantial preclinical evidence has illuminated metformin’s capacity to inhibit tumor growth across various cancers. This effect is primarily mediated by the activation of AMPK, a key regulator of cellular energy homeostasis. AMPK not only influences glucose and lipid metabolism but also directly impacts tumor bioenergetics by inhibiting the mammalian target of rapamycin (mTOR) pathway, which governs cell growth and proliferation [20]. This inhibition occurs through phosphorylation targeting either the raptor subunit of mTOR or tuberous sclerosis complex 2 (TSC2), while mTOR degradation is enhanced by unc-51-like kinase 1 (ULK1), an autophagy regulator. Additionally, AMPK promotes the phosphorylation and degradation of proteins like p70 ribosomal protein S6 kinase (p70S6K), critical for mRNA translation, further inhibiting the mTOR pathway [21,22]. Moreover, AMPK upregulation disrupts insulin signaling by reducing insulin receptor substrate-1 (IRS-1) phosphorylation, interrupting the phosphatidylinositol 3-kinase/protein kinase B (PI3K/Akt)/mTOR pathway, a known oncogenic driver [23]. While AMPK activation plays a central role in metformin’s anti-cancer effects, research suggests that metformin can also provide AMPK-independent anti-tumor benefits.

In breast cancer, metformin reduces tumor cell proliferation, partly by activating AMPK and inhibiting key elements like phosphorylated 4E-BP1 in the mTOR pathway. It also downregulates cyclin D1, halting cell cycle progression, and suppresses cyclooxygenase-2 (COX-2) expression, lowering the risks of lymphatic and distant metastasis [24]. Metformin also showed promise in ER^+^ tumors by increasing cell survival under estrogen-deprived conditions and enhancing mitochondrial respiration via fatty acid oxidation, especially when used alongside hormone therapies [25]. The drug exerts a profound impact on CAFs within the TME by disrupting pro-tumorigenic reprogramming processes. Specifically, metformin inhibits hypoxia-inducible factor-1 alpha (HIF-1a) expression through the activation of phosphorylated AMPK (p-AMPK). This suppression is associated with a reduction in the expression of pro-inflammatory cytokines, such as interleukin-8 (IL-8), which are governed by HIF-1α signaling and play critical roles in facilitating cancer progression. The attenuation of HIF-1α expression and its downstream effects further suggests a potential modulation of glucose metabolism in CAFs, as HIF-1α is a central regulator of glycolytic pathways under hypoxic conditions [26].

Metformin affects glucose metabolism, lowers lactate production, and improves tumor vascularization, contributing to a more immune-permissive TME by increasing CD8^+^ T cell infiltration [27,28]. Notably, metformin reprograms tumor-associated macrophages (TAMs) from the tumor-promoting M2 phenotype to the anti-tumorigenic M1 phenotype, thereby reducing angiogenesis and enhancing immune-mediated tumor suppression. This reprogramming is mediated through the activation of AMPK, which modulates macrophage polarization by interacting with the nuclear factor kappa-light-chain-enhancer of activated B cells’ (NF-κB) signaling pathway. Metformin’s upregulation of AMPK and its direct inhibition of mitochondrial Complex I activity may influence metabolic processes, such as glycolysis and oxidative phosphorylation (OXPHOS), favoring the metabolic profile associated with M1 polarization [29]. Other immune effects include boosted NK cell cytotoxicity and decreased immunosuppressive activity by myeloid-derived suppressor cells (MDSCs) [30]. These TME-modulating effects hold potential therapeutic benefits across various cancers characterized by an aggressive TME, including triple-negative breast cancer (TNBC) and colorectal cancer (CRC) [28,29,30,31].

Metformin also shows potential in treating other gynecological malignancies beyond breast cancer. In endometrial cancer, AMPK activation upregulated Ten-Eleven Translocation 2 methylcytosine dioxygenase 2 (TET2) gene expression, which inhibited cancer cell growth [32]. In cervical cancer, metformin’s upregulation of zinc finger protein 36 (ZFP36), an anti-tumor gene associated with mTOR complex1 (mTORC1) suppression, highlights its regulatory potential beyond AMPK [33]. In ovarian cancer, metformin influenced the AMPK/glycogen synthase kinase-3β (GSK3β) axis, leading to cyclin D1 degradation and G1 phase cell cycle arrest, with potential epigenetic effects under normoglycemic conditions [34,35]. Additionally, metformin disrupted transforming growth factor-β (TGF-β) signaling between ovarian cancer cells and mesothelial cells, reducing pro-inflammatory signals, including IL-8, and HIF1α-driven invasion [36].

In non-small cell lung cancer (NSCLC), metformin reduced proliferation by downregulating key oncogenic markers like hairy and enhancer of split-1 (HES1) and regulated the development and DNA damage response 1 (REDD1) by modulating p-mTOR and p53 levels [37]. Similar metabolic reprogramming effects were observed in pancreatic and prostate cancers, where metformin, through AMPK and peroxisome proliferator-activated receptor gamma coactivator-1alpha (PGC-1α) upregulation, enhanced mitochondrial function, while curtailing glycolysis and tumor growth [38,39]. Additional AMPK/mTOR-independent anti-cancer mechanisms included increased ROS production, disrupted OXPHOS, and induced redox imbalance, promoting apoptosis, as observed in prostate cancer models [40].

In hepatocellular carcinoma (HCC), metformin disrupted lipid metabolism, facilitated apoptosis, and bolstered oxidative stress through p38 mitogen-activated protein kinase (p38MAPK) activation [41], while its anti-metastatic potential, even at low doses, was underscored by the inhibition of the AMPK, c-Jun N-terminal kinase (JNK), IL-8, and the matrix metalloproteinase (MMP) 9 signaling axis [42]. A zebrafish model of metabolic dysfunction-associated fatty liver disease (MAFLD) and HCC demonstrated that a high-fat diet (HFD) promotes liver cancer, angiogenesis, and immune dysregulation, which metformin reversed by altering macrophage polarization and restoring T cell density [43]. Similar benefits were observed in cholangiocarcinoma (CCA), where metformin altered tumor metabolism by reducing lactate production and downregulating lactate dehydrogenase A (LDHA) expression, thereby disrupting the Warburg effect. This phenomenon describes the preference of cancer cells for glycolysis as their primary energy source, even under normal oxygen conditions, which supports tumor growth and survival [44].

In recent years, spatial transcriptomics has emerged as a cutting-edge technique for evaluating drug effects on cancer outcomes, allowing the detailed mapping of gene expression within tumor tissues. This innovative approach enables researchers to dissect the intricate interactions within the TME, including the cellular and molecular changes induced by therapeutic agents. Notably, spatial transcriptomics was employed to evaluate the impact of metformin in cancer [45,46]. In pancreatic cancer, for instance, metformin was linked to improved survival and immune modulation, reducing pro-tumoral macrophage activity and enhancing anti-tumor DC function. This translated into significantly better overall survival and five-year survival rates among metformin users in the PREOPANC trial [45]. Additionally, transcriptomic studies in CRC revealed that metformin modulates miRNA interactions that target critical growth pathways, particularly PI3K/Akt and MAPK/extracellular signal-regulated kinase (ERK), highlighting its potential for the post-transcriptional regulation of cancer progression [46].

**Figure 1:**
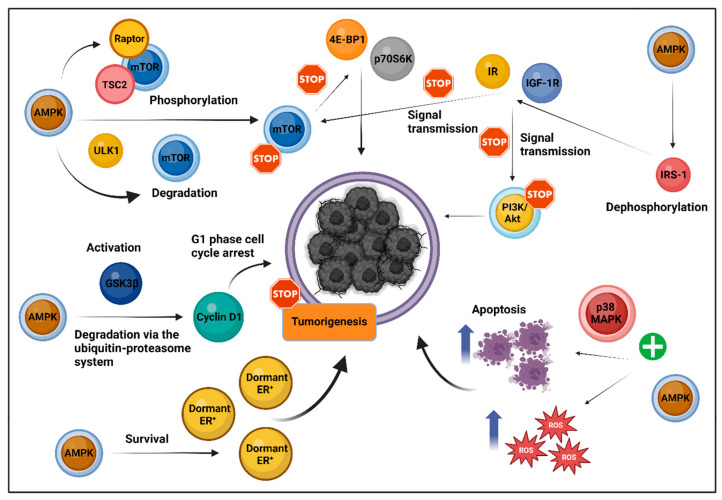
The impact of metformin on cancer-related signaling pathways via AMPK activation. AMPK activation by metformin disrupts key cancer-related signaling pathways, leading to inhibition of tumor growth and proliferation. AMPK deactivates the mTOR signaling pathway through phosphorylation and degradation mechanisms, inhibiting proteins such as p70S6K and 4E-BP1 involved in mRNA translation, thereby limiting cancer cell proliferation. Additionally, AMPK reduces phosphorylation of IRS-1, impairing signal transmission from IRs and IGF-1Rs, which disrupts the PI3K/Akt/mTOR signaling axis. In breast cancer, AMPK activation supports the survival of dormant ER^+^ tumor cells under low-estrogen conditions. In ovarian cancer, AMPK influences the AMPK/GSK3β axis, triggering cyclin D1 degradation through the ubiquitin-proteasome system, resulting in cell cycle arrest at the G1 phase. AMPK activation also promotes the production of ROS and induces apoptosis, which collectively hinder tumor growth [20,22,23,25,34,41]. Abbreviations: 4E-BP1: Eukaryotic translation initiation factor 4E-binding protein 1; Akt: Protein kinase B; AMPK: AMP-activated protein kinase; ER^+^: Estrogen receptor-positive; GSK3β: Glycogen synthase kinase 3 beta; IGF-1R: Insulin-like growth factor receptor; IR: Insulin receptor; IRS-1: Insulin receptor sub-strate-1; mTOR: Mammalian target of rapamycin; mRNA: Messenger RNA; p38MAPK: p38 mitogen-activated protein kinase; p70S6K: p70 ribosomal protein S6 kinase; PI3K: Phosphoinositide 3-kinase; Raptor: Regulatory-associated protein of mTOR; ROS: Reactive oxygen species; TSC2: Tuberous sclerosis complex 2; ULK1: Unc-51-like autophagy activating kinase 1. Created with www.BioRender.com.

Despite the diverse mechanisms through which metformin positively affects carcinogenesis, the activation of AMPK is the central mechanism of its action. Figure 1 presents significant AMPK-mediated pathways that contribute to metformin’s broad anti-cancer potential.

### 2.2. Insulin Therapy and Sulfonylureas

#### 2.2.1. Insulin Therapy

A robust body of experimental evidence notes insulin’s role in fostering a pro-tumorigenic environment through its modulation of key cancer-associated signaling pathways. One prominent mechanism involves the mTOR pathway, where insulin enhances glucose uptake and glycogenolysis, processes that fuel cancer cell growth [47]. Additionally, insulin activates the IRS/PI3K/Akt and MAPK pathways, which govern cell cycle progression, anti-apoptotic responses, and angiogenesis [48]. Angiogenesis, the formation of new blood vessels, is particularly crucial for tumor progression, as it supplies essential nutrients and oxygen to the expanding tumor mass. Insulin’s influence on these signaling pathways has been linked to a heightened potential for carcinogenesis across various cancer types. For instance, insulin promoted glioblastoma (GB) cell proliferation and survival through the activation of the Akt pathway [49]. Evidence indicates that both human insulin and insulin glargine can stimulate thyroid cell proliferation at elevated concentrations, increasing the phosphorylation of Akt and ERK1/2 in a dose-dependent manner, with insulin glargine showing prolonged effects. Notably, therapeutic doses of insulin did not appear to stimulate cell proliferation [50]. In lung cancer, insulin facilitated cell proliferation and migration by upregulating the PI3K/Akt pathway [51]. Similarly, in HCC, insulin enhanced cell growth and survival through the activation of both the PI3K/Akt and Rat sarcoma (Ras)/MAPK pathways [52]. Notably, in CRC, insulin drove tumor progression and metastasis by upregulating acetyl-CoA acetyltransferase 1 (ACAT1), an enzyme critical for cancer progression through its role in lipid metabolism and mitochondrial function, thereby supporting cancer cell growth, survival, and metastatic potential [53].

Existing evidence indicates that hyperinsulinemia plays a role in the progression of pancreatic cancer, as demonstrated by Zhang et al., who found that genetically reducing insulin production in mice led to an approximate 50% reduction in pancreatic intraepithelial neoplasia (PanIN) lesions [54]. Their research further suggests that managing insulin levels could inhibit pancreatic cancer by altering various cell types within the pancreatic microenvironment. Specifically, modifying *Ins2* gene dosage in a mouse model lacking the rodent-specific *Ins1* gene modestly reduced hyperinsulinemia across genders without substantially affecting glucose tolerance. This reduction in insulin was associated with fewer PanIN and acinar-to-ductal metaplasia lesions. Single-cell transcriptomics revealed that hyperinsulinemia influences several pancreatic cell types, particularly immune cells, by modifying protein synthesis pathways and key signaling cascades such as MAPK/ERK and PI3K/Akt [55].

In addition to these direct signaling effects, hyperinsulinemia can indirectly drive carcinogenesis, particularly in the context of obesity, a condition often accompanied by both hyperinsulinemia and insulin resistance. Obesity-related hyperinsulinemia leads to an imbalance in angiogenic factors, elevating pro-angiogenic agents, such as vascular endothelial growth factor (VEGF) and leptin, while reducing anti-angiogenic factors like adiponectin. This imbalance fostered a tumor-supportive microenvironment that promoted cancer progression and metastasis [56]. Moreover, chronic hyperinsulinemia arising from both endogenous overproduction and exogenous insulin administration was linked to persistent chronic low-grade inflammation. Elevated inflammatory cytokines, including IL-6 and tumor necrosis factor-alpha (TNF-α), played pivotal roles in creating an immunosuppressive, pro-angiogenic environment that further accelerated tumor growth and metastasis [57].

Insulin-like growth factor (IGF) signaling also plays a crucial role in cancer development. Due to the structural similarity between insulin and IGF-1, elevated insulin levels under hyperinsulinemic conditions can activate the IGF-1 receptor (IGF-1R), amplifying mitogenic and anti-apoptotic effects. Both insulin and IGF-1R signaling activated downstream pathways that regulate cell growth, proliferation, survival, and metabolism, processes that can be exploited by cancer cells to sustain tumor development [58]. Experimental models demonstrated that disrupting insulin receptor (IR) signaling, through either genetic manipulation or pharmacological inhibition, can significantly reduce tumor growth and metastasis. For example, in mouse models of mammary carcinoma, hyperinsulinemia increased tumor growth and metastasis, while treatment with insulin sensitizers or IR/IGF-1R inhibitors mitigated these effects. Likewise, silencing the IR in breast cancer cells reduced their metastatic potential [59].

Notably, concerns have been raised regarding the potential cancer-promoting effects of insulin analogs, particularly long-acting formulations such as insulin glargine. Due to structural modifications, these analogs may exhibit altered affinities for IR and IGF-1R, possibly enhancing mitogenic signaling. While in vitro studies suggested insulin glargine may have greater mitogenic potency compared to regular insulin, in vivo data remain inconclusive, and no definitive conclusions about its carcinogenic risk were established [60].

#### 2.2.2. Sulfonylureas

Experimental studies on the anti-cancer efficacy of sulfonylureas (SUs) have shown encouraging results, with glibenclamide emerging as one of the most thoroughly studied SUs. In various preclinical models, glibenclamide has consistently demonstrated notable anti-cancer effects. For instance, in breast cancer cell lines, glibenclamide induced cytostatic effects through the modulation of adenosine triphosphate (ATP)-sensitive potassium channels, resulting in cell cycle arrest at the G0/G1 phase. This process is associated with the upregulation of the cyclin-dependent kinase inhibitor p27 and downregulation of cyclin E, thereby inhibiting tumor growth [61].

Glibenclamide may also influence carcinogenesis by altering the TME, particularly in NSCLC. A recent study by Chen et al. found that glibenclamide inhibited the conversion of normal fibroblasts into CAFs by blocking sulfonylurea receptor 1 (SUR1) and upregulating let-7a-5p, a microRNA that inhibits the fibroblast-to-CAF transition by targeting TGF-β signaling. The transformation of normal fibroblasts into CAFs is often associated with metabolic reprogramming, including a shift toward glycolysis that supports rapid cell proliferation and biomass accumulation. Since CAFs promote tumor growth, angiogenesis, and immune evasion, their prevention can foster a more immune-permissive TME and enhance T lymphocyte activity against tumor cells [62].

In HCC, glibenclamide showed inhibitory effects on voltage-gated potassium (Kv) channels, leading to dose- and calcium-dependent reductions in cell adhesion and proliferation [63]. Prostate cancer models similarly showed glibenclamide’s capacity to induce dose-dependent growth inhibition and apoptosis within hours of administration [64]. Additionally, glibenclamide impacted angiogenesis and metastasis; in ovarian cancer, it reduced cellular invasion and migration by suppressing the secretion of platelet-derived growth factor-AA (PDGF-AA), an essential factor in tumor angiogenesis [65].

Other SUs have also exhibited anti-tumor effects. Chlorpropamide and gliclazide, for instance, inhibited TNF-α production, a cytokine crucial for cell survival and apoptosis regulation. Glipizide was found to reduce tumor growth, angiogenesis, and metastasis in breast cancer and melanoma models [66,67]. Additionally, acetohexamide demonstrated efficacy in preventing breast cancer cell entry into lymphatic ducts, further suggesting a role for SUs in reducing metastasis [68].

### 2.3. Pioglitazone

Pioglitazone has also garnered attention for its complex role in cancer biology, particularly concerning urogenital malignancies. Bladder cancer remains a major issue in the context of pioglitazone therapy, with experimental evidence indicating that pioglitazone may induce DNA damage and malignant transformation in bladder cells, affecting gene expression and epithelial–mesenchymal transition (EMT). EMT is a crucial biological process in which epithelial cells lose their adhesion and polarity while acquiring mesenchymal properties, such as enhanced migratory capacity and invasiveness, significantly contributing to the progression and aggressiveness of many cancers [69]. This is linked to the activation of PPAR-γ signaling that further promotes de novo lipogenesis, potentially supporting tumor growth [70]. However, contradictory findings complicate this narrative, as recent data indicate that pioglitazone may actually protect against bladder cancer progression by downregulating oncogenic markers such as p53 and cyclin D1, without promoting malignant transformations in normal bladder cells [71]. Importantly, these observations are further supported by proteomic analyses [72]. Beyond bladder cancer, pioglitazone’s impact on other urogenital malignancies, including kidney and prostate cancers, remains contentious. In prostate cancer, pioglitazone may reduce tumorigenesis by alleviating obesity-related inflammation [73], while in renal cell carcinoma (RCC), the drug induced apoptosis through caspase activation and the downregulation of anti-apoptotic proteins [74].

Research also extended pioglitazone’s potential to a variety of other cancers. In breast cancer, it inhibited cell proliferation and migration, likely through modulation of the Janus kinase/signal transducer and activator of transcription 3 (JAK/STAT3) signaling pathway, while also increasing adiponectin levels [75]. In lung cancer, combining pioglitazone with celecoxib was shown to reduce tumor weight and improve survival by inhibiting NF-κB-mediated proliferation [76]. In NSCLC, pioglitazone targeted cancer cell metabolism, reducing extracellular acidification and glucose metabolism markers, thus limiting cancer cells’ ability to adapt to the nutrient-poor, hypoxic TME [77].

Despite these promising results, preliminary evidence suggests that PPAR-γ activation may have a dual effect on tumor progression. It can exert anti-tumorigenic effects on cancer cells while promoting pro-tumorigenic outcomes within the TME, particularly in myeloid cells. For example, Li et al. demonstrated that although PPAR-γ activation inhibited cancer cell growth, it paradoxically promoted metastasis in lung cancer models. In two mouse models, pioglitazone facilitated metastasis to the liver, brain, and lungs by increasing the accumulation of pro-tumor arginase I-positive macrophages. This metastasis-promoting effect was shown to be PPAR-γ dependent in myeloid cells, as mice with myeloid-specific PPAR-γ deletion exhibited reduced metastatic spread [78].

Pioglitazone also exhibited anti-fibrotic and hepatoprotective effects in HCC models, acting through pathways such as the MAPK and AMPK [79]. In pancreatic cancer, pioglitazone reduced metastasis by altering inflammation-related gene expression, including CEA and COX-2 [80]. Additionally, it enhanced ROS formation and suppressed B cell lymphoma 2 protein (Bcl-2) expression in hypoxic cancer cells, highlighting its potential role in controlling metastasis [81]. In CRC, pioglitazone reduced cancer stem cell viability and promoted a mesenchymal-to-epithelial transition (MET) [82]. Moreover, pioglitazone influenced the immune landscape of the TME in CRC by inhibiting the IL-6/STAT3 pathway, a vital regulator of immune evasion. Its downregulation, reflected in reduced IL-6 mRNA and STAT3 activity, enhanced mature DC functionality, boosting CD8^+^ T cell priming and increasing tumor-infiltrating lymphocytes (TILs). Pioglitazone also amplified T cell cytotoxicity, significantly reducing tumor cell viability. By targeting metabolic and inflammatory barriers to DC activity, it strengthened anti-tumor responses, as evidenced by elevated IL-12 and interferon-gamma (IFN-γ) levels in combination-treated groups [83].

In hematological cancers, pioglitazone showed promise in enhancing anti-leukemic effects by inhibiting the hyperactivated PI3K/Akt pathway [84]. In chronic myeloid leukemia (CML), it disrupted CD44, a surface protein essential for cancer stem cell adhesion and migration within the TME. By inhibiting CD44, pioglitazone reduced the invasiveness of CML cells, potentially limiting metastasis and disease progression [85].

### 2.4. Incretin Mimetics

#### 2.4.1. DDP-4 Inhibitors

Despite their benefits in managing glycemic levels, DPP-4 inhibitors demonstrated inconsistent results in cancer research, likely due to the significant heterogeneity in DPP-4 expression across different tumor types and environments [86]. In breast cancer, DPP-4 inhibition was linked to enhanced metastasis through the activation of the C-X-C motif chemokine ligand 12/C-X-C chemokine receptor type 4 (CXCL12/CXCR4)/mTOR signaling, a pathway known to promote EMT [87]. This metastatic propensity was further exacerbated by the activation of the ROS–(nuclear factor erythroid 2-related factor 2)Nrf2–heme oxygenase-1 (HO-1) axis, which led to NF-κB activation and upregulation of inflammatory cytokines, such as IL-6. This pro-inflammatory response is thought to create an environment conducive to tumor progression by supporting angiogenesis and suppressing immune activity [88]. Notably, saxagliptin was associated with increased migratory and invasive capabilities in thyroid cancer cells via Nrf2 upregulation, which may synergize with oncogenic pathways like mTOR and heat shock factor 1 (HSF1) to promote cancer cell survival and spread [89]. Additionally, animal studies raised concerns regarding DPP-4 inhibitors’ role in pancreatic β-cell proliferation, a process that could potentially lead to neoplastic transformation within the pancreatic tissue [90].

Conversely, in other cancer contexts, DPP-4 inhibition may impart anti-tumor benefits, particularly by countering CAF-driven growth. In scirrhous gastric cancer (SGC), DPP-4 released from CAFs was identified as a key factor enhancing cancer cell proliferation, likely through CXCR4 signaling [91]. Similarly, in endometrial cancer, sitagliptin was observed to limit proliferation, invasion, and tumorigenesis [92], while in CRC models, DPP-4 inhibition reduced cell proliferation, inhibited angiogenesis, and promoted apoptosis [93]. Notably, in CRC, DPP-4 may exert both tumor-promoting and tumor-suppressive effects, depending on its expression levels and the specific context within the TME. Elevated CD26 levels, the active form of DPP-4, were associated with a poor prognosis and enhanced metastatic potential in CRC patients [94].

Additionally, DPP-4 inhibition may enhance anti-tumor immunity through the recruitment of eosinophils, which play a critical role in tumor cell death and the amplification of anti-tumor immune responses within the TME. Research indicates that, by preserving chemokines like C-C motif chemokine ligand (CCL)11, sitagliptin promoted eosinophil infiltration, an effect seen in models of HCC and breast cancer. This eosinophilic migration is IL-33 dependent and synergizes with immune checkpoint therapies, suggesting a novel mechanism whereby DPP-4 inhibition can bolster eosinophil-driven anti-tumor responses [95]. DPP-4 inhibition may also play a therapeutic role in obesity-driven HCC. Elevated DPP-4 levels associated with a HFD were found to drive HCC growth, metastasis, and angiogenesis via CCL2 signaling. In both cell and animal models, DPP-4 inhibition through vildagliptin or genetic deletion reduced HCC progression, effectively disrupting the pro-angiogenic DPP-4/CCL2 pathway. Clinical data further underscored these findings, showing that higher serum DPP-4 levels correlated with a worse prognosis in HCC patients [96]. Intriguingly, DPP-4 inhibition may enhance anti-tumor immune responses by improving the functionality of type 1 conventional dendritic cells (cDC1s). Sitagliptin, for example, enhanced antigen presentation by cDC1s, thereby facilitating T cell activation and subsequent tumor suppression. Mechanistically, sitagliptin appeared to stabilize key signaling molecules essential for cDC1 activity and may help restore glucose availability, thus addressing the metabolic requirements of these immune cells in the TME [97].

#### 2.4.2. GLP-1 and Dual GIP/GLP-1 Receptor Agonists

GLP-1 RAs demonstrated varied effects in cancer, with concerns remaining about their impact on thyroid and pancreatic malignancies. Notably, the use of GLP-1 RAs is associated with an increased incidence of medullary thyroid cancer (MTC). Animal studies indicated that GLP-1 agonists such as exenatide and liraglutide elevated calcitonin levels and induced C-cell hyperplasia in rodent models [98,99]. However, the implications for human health remain uncertain, as GLP-1 receptor expression in human C-cells was minimal [100]. Interestingly, recent evidence demonstrated liraglutide’s inhibitory effects on cell growth and migration in both papillary and medullary thyroid cancers by modulating the PI3K/Akt/mTOR pathway in a dose- and time-dependent manner [101].

Concerns also extend to the association between GLP-1 RAs and pancreatic cancer, as chronic pancreatitis, a potential side effect of GLP-1 RAs, is linked to an increased risk of malignancy [102]. However, liraglutide was found to inhibit pancreatic cancer growth and promote apoptosis by suppressing the PI3K/Akt and ERK1/2 signaling pathways [103]. Furthermore, GLP-1 agonism can modulate calcium signaling and cadherin expression, which are crucial for cell adhesion and mobility, thereby inhibiting the proliferation and migration of pancreatic cancer cells [104]. Additionally, GLP-1 RAs demonstrated protective effects on pancreatic β-cells in hyperglycemic states, potentially reducing apoptosis through the modulation of miR-139-5p and IRS-1 suppression [105].

In addition to thyroid and pancreatic cancers, the impact of GLP-1 RAs was explored in various other malignancies, with experimental studies revealing promising outcomes. For instance, exenatide inhibited glioma cell migration and invasion via the GLP-1R/sirtuin 3 (SIRT3) pathway [106], while in breast cancer, it reduced proliferation and promoted apoptosis by inhibiting NF-κB nuclear translocation [107]. Notably, liraglutide significantly debilitated breast cancer cell growth in the presence of a conditioned medium from obese adipose tissue, implicating adipokines in its mechanism [108]. However, at high concentrations, liraglutide may facilitate the progression of TNBC through the NADPH oxidase 4 (NOX4)/ROS/VEGF signaling pathway, highlighting the importance of dosage and cancer phenotype [109]. Similar beneficial effects in breast cancer were observed with dulaglutide, which demonstrated its anti-tumor properties by reactivating tumor suppressor genes through promoter demethylation [110].

The activation of AMPK by GLP-1 RAs further contributed to reduced cancer cell viability, particularly in glycolytic metabolism [111]. In endometrial carcinoma, both liraglutide and tirzepatide may suppress cell proliferation and exhibit anti-tumor effects, respectively, with tirzepatide exhibiting essential anti-obesity and anti-tumorigenic effects, potentially modulating metabolic and immune pathways relevant to tumor growth [112,113]. Research also suggests that exenatide may reduce the aggressiveness of ovarian cancer, as it appeared to modulate key cellular behaviors, including reducing cancer cell migration and promoting apoptosis through caspase activation, thereby directly influencing cancer cell dynamics within the TME. Furthermore, it modulated extracellular matrix remodeling, a vital factor in metastasis, by regulating the expression of MMP-2 and MMP-9 and their tissue inhibitors (TIMP-1 and TIMP-2). Additionally, exenatide decreased the production of adhesion molecules, such as intercellular adhesion molecule-1 (ICAM-1) and vascular cell adhesion molecule-1 (VCAM-1), limiting cancer cell adherence to the endothelial lining and thus inhibiting metastatic spread. In TNF-α-stimulated endothelial cells, the reduction in MMP-1 and MMP-9 production suggests that GLP-1 RAs may alter the TME’s capacity to facilitate cancer cell invasion and spread [114].

Both liraglutide and semaglutide showed potential in preventing disease progression and reducing tumor burden in HCC [115,116]. GLP-1 RAs also offer potential benefits for immune functionality, particularly by reversing obesity-induced impairments in NK cell activity. Preclinical data indicated that liraglutide enhanced NK cell-mediated cytotoxicity against HCC cells, independently of CD8^+^ T cells. Its anti-cancer effects were tied to the inhibition of the IL-6/STAT3 axis, often linked with unchecked cellular proliferation and immune evasion mechanisms. Beyond immune modulation, liraglutide likely impacted the TME’s metabolic landscape, potentially enhancing anti-cancer immune responses by modulating metabolic pathways such as glycolysis, thereby strengthening NK cell-mediated tumor suppression [117]. Similar immunomodulatory effects were also observed with semaglutide. Clinical findings by De Barra et al. demonstrated that six months of semaglutide therapy in obese patients enhanced NK cell cytotoxicity, increasing the production of interferon-γ and granzyme B, both markers of NK cell activity. This improvement was linked to activation of the CD98-mTOR-glycolysis axis, a pivotal pathway for NK cell cytokine production, and notably, these enhancements in NK cell function were independent of weight reduction [118].

Furthermore, GLP-1 RAs inhibited cell cycle progression and promoted apoptosis in CRC via the PI3K/Akt/mTOR pathway [119]. Preliminary evidence also suggested GLP-1 receptor expression in metastatic castrate-resistant prostate cancer, indicating opportunities for further research in urinary tract malignancies [120]. Genetic evidence further outlined the role of GLP-1 signaling in cancer immunology. Zhu et al. examined GLP-1 signaling-related genes across 33 cancer types, identifying significant associations between GLP-1 signaling and immune cell infiltration levels, including CD4^+^ T cells, NK cells, neutrophils, DCs, and macrophages. A lower GLP-1 signaling score, derived from gene enrichment analysis, was correlated with reduced immune cell infiltration, poorer survival rates, and lower responsiveness to immunotherapy across multiple cancer types. In CRC specifically, semaglutide reduced cellular migration, suggesting additional applications of GLP-1 RAs in impeding cancer spread [121].

### 2.5. SGLT-2 Inhibitors

A growing body of experimental research has highlighted the significant overexpression of SGLT-2 in malignant cells across various cancer types. Evidence suggests that targeting SGLT-2 can hinder tumor progression by disrupting the metabolic and signaling pathways critical for cancer cell survival. However, current data face challenges in clearly defining the efficacy of SGLT-2 inhibition across different malignancies, primarily due to notable differences in their metabolic pathways. The metabolism of each SGLT-2 inhibitor involves distinct enzymatic processes that affect drug inactivation and excretion, influencing stability, duration, and, ultimately, therapeutic outcomes. These metabolic discrepancies complicate efforts to establish universal conclusions regarding the anti-cancer potential of SGLT-2 inhibition. Nevertheless, SGLT-2 inhibition remains a promising therapeutic approach, demonstrating groundbreaking results in several malignancies, as supported by numerous experimental studies [122].

For instance, in glioblastoma, canagliflozin inhibited cell proliferation and glucose uptake, especially at 40 μM concentrations. Mechanistically, canagliflozin promoted AMPK phosphorylation while suppressing p70 S6 kinase and S6 ribosomal protein, both fundamental in cancer growth pathways [123]. In the context of thyroid cancer, canagliflozin showed promise in modulating the TME by downregulating pro-tumor chemokines such as CXCL8 and CCL2. By suppressing these chemokines, canagliflozin limited inflammatory cell recruitment into the TME, potentially reducing immune suppression and enhancing anti-tumor immunity. Canagliflozin also affected the survival and proliferation of both cancerous and endothelial cells, reducing IL-6 levels while increasing CXCL10 expression, indicating a shift toward an anti-tumor TME that may enhance immune response against cancer cells [124].

Contrary to concerns of breast cancer promotion by SGLT-2 inhibitors, experimental data suggest beneficial outcomes. Recent findings suggest that SGLT-2 inhibitors may offer a novel approach for targeting glucose metabolism in the TME of breast cancer. By restricting glucose availability, SGLT-2 silencing not only inhibited tumor cell proliferation but may also alleviate immunosuppressive conditions associated with high-glucose states, thus enhancing immune cell functionality. Mathematical models indicated that the optimal dosing of SGLT-2 inhibitors can reduce or eradicate tumor cells while sparing normal cells [125]. Novel insights revealed promising therapeutic effects of specific SGLT-2 inhibitors. Dapagliflozin, for example, demonstrated effectiveness in reducing breast cancer cell viability and triggering early apoptosis, primarily through the modulation of glucose metabolism. Likewise, empagliflozin exhibited anti-inflammatory properties that may influence tumor progression by altering the metabolic profile of the TME [126]. Moreover, empagliflozin acted as an miR-128-3p mimic, reducing CD44 expression under hypoxic conditions and fostering differentiation. In vivo, it inhibited tumor growth, limited lung metastasis, and elevated oxidative stress markers, potentially sensitizing cells to ferroptosis [127,128]. Data further indicated that SGLT-2 expression was significantly elevated in estrogen-sensitive breast cancer cells but remained undetectable in normal mammary tissue. The inhibition of SGLT-2 by ipragliflozin led to decreased cell proliferation and DNA synthesis, an effect negated by SGLT-2 knockdown. This mechanism appeared to involve disrupted glucose and sodium transport, resulting in reduced intracellular sodium influx, membrane hyperpolarization, mitochondrial destabilization, and the subsequent inhibition of cancer cell growth [129].

Moreover, SGLT-2 inhibition showed promise in HCC, with Shiba et al. demonstrating that canagliflozin mitigated metabolic dysfunction-associated steatohepatitis (MASH)-related HCC in a Western diet-fed mouse model by reducing hepatic steatosis, fibrosis, and tumor occurrence while enhancing adipose tissue health, promoting a “healthy expansion” profile marked by a lower oxidative stress index [130]. In HCC, tumor progression was closely linked to the WNT/β-catenin signaling pathway’s enhancement of aerobic glycolysis in malignant cells. Hung et al. reported that canagliflozin inhibited glucose uptake by targeting multiple glucose transporters, including GLUT1, thereby reducing HCC cell survival and colony formation. This effect was achieved through the disruption of β-catenin signaling, promoting its proteasomal degradation and inhibiting dephosphorylation, ultimately impairing tumor growth and extending survival [131]. HCC, characterized by metabolic reprogramming, hypoxia, and dysregulated signaling pathways, depends on hypoxia to drive tumor progression through angiogenesis, EMT, and glycolysis. Within the hypoxic TME of HCC, canagliflozin disrupted pro-tumor pathways by downregulating VEGF. Additionally, it suppressed glycolysis-associated proteins and EMT processes, essential for tumor progression and metastasis. By targeting the Akt/mTOR pathway, canagliflozin inhibited the accumulation of HIF-1α, reducing the metabolic and signaling adaptations critical for tumor survival and proliferation [132]. Similarly, in NSCLC, canagliflozin disrupted HIF-1α stabilization, interfering with mitochondrial function and survival pathways through mTOR inhibition and histone deacetylase 2 (HDAC2) suppression [133].

In gastric cancer, dapagliflozin reduced tumor proliferation by downregulating OTU deubiquitinase 5 (OTUD5), leading to yes-associated protein 1 (YAP1) degradation, a key protein in tumor progression [134]. Canagliflozin also demonstrated efficacy in CRC, disrupting cellular metabolism and inducing endoplasmic reticulum (ER) stress, which promotes autophagy and apoptosis through the upregulation of SIRT3 [135]. In pancreatic cancer models, canagliflozin’s inhibition of the PI3K/Akt/mTOR pathway highlighted its anti-tumor potential by hindering glycolysis [136].

**Figure 2:**
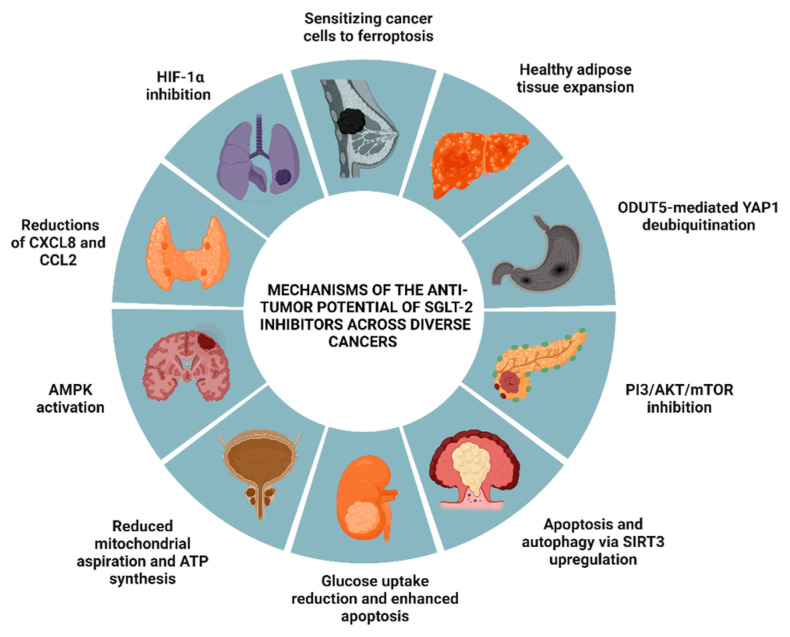
Anti-tumor activity of SGLT-2 inhibitors in cancer models: insights from animal studies [123,124,125,127,130,133,136,137,138]. Abbreviations: AMPK: AMP-activated protein kinase; ATP: Adenosine triphosphate; CCL2: C-C motif chemokine ligand 2; CXCL8: C-X-C motif chemokine ligand 8; HIF-1α: Hypoxia-inducible factor 1-alpha; mTOR: Mammalian target of rapamycin; ODUT5: OTU deubiquitinase 5; PI3K: Phosphoinositide 3-kinase; SGLT-2: Sodium-glucose co-transporter 2; SIRT3: Sirtuin 3; YAP1: Yes-associated protein 1. Created with www.BioRender.com.

Investigations into urinary tract cancers showed that dapagliflozin exerted cytotoxic effects in RCC by reducing glucose uptake, regulating cell cycle progression, and promoting apoptosis in RCC cells [137]. Notably, canagliflozin also reduced cell growth in prostate cancer by curtailing mitochondrial respiration and ATP production, which parallels the metabolic effects seen with metformin. This mechanism involves AMPK activation, reduced lipid synthesis, and limited glucose uptake [138]. Despite clinical concerns about bladder cancer risks with SGLT-2 inhibitors, preclinical findings generally showed no increased bladder cancer incidence with dapagliflozin, even at high doses [139]. Comparable benefits were also documented in rarer malignancies, such as osteosarcoma, where SGLT-2 inhibition appeared to activate the stimulator of interferon genes (STING) pathway, subsequently upregulating interferon regulatory factor 3/interferon-beta (IRF3/IFN-β) signaling. The activation of this pathway within the TME enhanced immune surveillance and tumor suppression, promoting interferon production and other immune-activating signals that facilitate immune cell recruitment to target cancerous tissues [140]. Figure 2 depicts the anti-tumor effects of SGLT-2 inhibitors across various cancer models, as demonstrated in animal studies.

**Figure 3:**
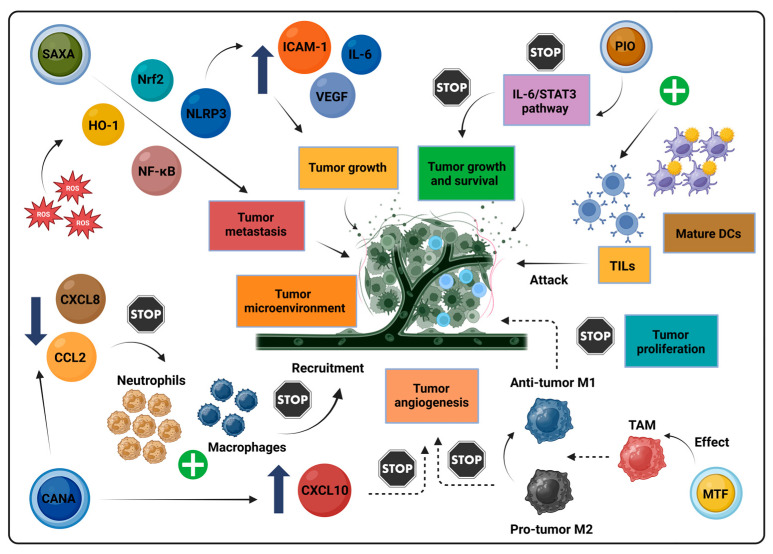
Schematic illustration of the diverse mechanisms through which anti-diabetic pharmacotherapy may affect tumor microenvironment dynamics. Metformin promotes the conversion of TAMs into an M1 phenotype, thereby enhancing anti-proliferative activity and inhibiting angiogenic processes. Pioglitazone modifies immune responses through the targeting of the IL-6/STAT3 signaling pathway, leading to increased activation of TILs, which are essential for recognizing and attacking tumor cells in the presence of mature dendritic cells. Additionally, canagliflozin reduces the release of the inflammatory chemokines CXCL8 and CCL2, which may limit the recruitment of immune cells while simultaneously increasing the chemokine CXCL10, ultimately inhibiting tumor angiogenesis. Conversely, DPP-4 inhibitors may promote metastatic processes by activating the ROS-mediated Nrf2/HO-1/NF-κB/NLRP3 signaling axis, resulting in elevated production of inflammatory cytokines, adhesion molecules, and angiogenic factors, including IL-6, ICAM-1, and VEGF [29,83,88,124]. Abbreviations: CANA: Canagliflozin; CCL2: C-C motif chemokine ligand 2; CXCL8: C-X-C motif chemokine ligand 8; CXCL10: C-X-C motif chemokine ligand 10; DCs: Dendritic cells; DPP-4: Dipeptidyl peptidase-4; HO-1: Heme oxygenase-1; ICAM-1: Intercellular adhesion molecule 1; IL-6: Interleukin 6; M1 phenotype: Macrophage 1 phenotype; M2 phenotype: Macrophage 2 phenotype; MTF: Metformin; NLRP3: NOD-like receptor family pyrin domain containing 3; NF-κB: Nuclear factor kappa-light-chain-enhancer of activated B cells; Nrf2: Nuclear factor erythroid 2-related factor 2; PIO: Pioglitazone; ROS: Reactive oxygen species; SAXA: Saxagliptin; STAT3: Signal transducer and activator of transcription 3; TILs: Tumor-infiltrating lymphocytes; TAMs: Tumor-associated mac-rophages; VEGF: Vascular endothelial growth factor. Created with www.BioRender.com.

Across various therapeutic classes, anti-diabetic medications exhibit the capacity to modulate cancer-related signaling pathways and impact the TME, with promising immunomodulatory effects. While metformin stands out with the most robust data supporting its anti-cancer potential, emerging research on newer agents, such as GLP-1 RAs and SGLT-2 inhibitors, is also encouraging, though still in its nascent stages. Conversely, evidence for DDP-4 inhibitors and pioglitazone remains mixed, showing potential benefits only in specific cancer contexts, and findings on exogenous insulin administration lack clarity. The divergent results around hyperinsulinemia’s malignancy-promoting effects and the complex role of insulin secretagogues, like SUs, further complicate the landscape. This complexity highlights the need for further research to fully understand how anti-diabetic treatments affect cancer biology and their potential therapeutic applications. Table 1 summarizes key molecular mechanisms by which anti-diabetic drugs influence cancer development and progression across various cancer types. Additionally, Figure 3 illustrates diverse mechanisms by which these agents may influence cancer development and progression through their effects on the TME.

## 3. Enhancing Chemotherapy: The Integration of Anti-Diabetic Treatments in Improving Therapeutic Outcomes

### 3.1. Exploring Anti-Diabetic Therapies as a Strategy to Diminish Chemotherapy-Induced Cardiotoxicity

Anthracycline-related cardiotoxicity, particularly linked to the widespread use of doxorubicin (DOX) in cancer treatment, remains a significant clinical challenge. Emerging evidence indicates that many anti-diabetic agents, known for their pleiotropic effects beyond glycemic control, may confer cardioprotective benefits. This has sparked growing interest in their potential role in mitigating DOX-induced cardiac damage, a protective effect demonstrated primarily in experimental studies and, to a lesser extent, in clinical settings.

#### 3.1.1. Metformin

Metformin demonstrates significant therapeutic potential in mitigating DOX-induced cardiotoxicity through its versatile effects on inflammation, oxidative stress, apoptosis, and mitochondrial dysfunction. Its anti-inflammatory properties are characterized by the downregulation of the high-mobility group box 1 (HMGB1)/NF-κB/NLRP3 signaling axis, alongside the modulation of AMPK and MAPK pathways, both of which play crucial roles in cellular stress responses [171,172]. Notably, AMPK upregulation can be achieved even at low doses [173], and metformin may also enhance AMPK activation indirectly by promoting the expression of adiponectin and its receptors [174]. In addition to its anti-inflammatory effects, metformin pre-treatment appears to confer protective benefits by restoring critical anti-oxidant enzyme activity, inhibiting caspase-mediated apoptosis, and reducing DNA fragmentation [175]. Furthermore, recent investigations underscored metformin’s ability to modulate autophagy and mitophagy pathways, normalizing the expression of essential autophagy-related proteins such as beclin-1, microtubule-associated protein 1A/1B light chain 3B (LC3B-II), and p62, which are pivotal for maintaining cellular homeostasis [176]. Comparable cardioprotective effects were documented in cases of trastuzumab-induced cardiotoxicity, which mirrors the cardiotoxic mechanisms associated with DOX [177]. Conversely, recent animal data failed to replicate the cardioprotective benefits of metformin in the context of cardiotoxicity stemming from combination chemotherapy regimens, including cyclophosphamide, methotrexate, and 5-fluorouracil (5-FU) [178].

A recent meta-analysis of animal studies underpinned the centrality of metformin’s anti-oxidative mechanisms in its cardioprotective role, providing further support for its potential in mitigating DOX-induced cardiotoxicity [179]. However, clinical evidence remains inconclusive. In a randomized controlled trial involving breast cancer patients, metformin did not prevent myocardial injury, as evidenced by elevated high-sensitivity troponin-I levels and reductions in left ventricular ejection fraction (LVEF). Nonetheless, metformin was observed to play a significant role in preserving mitochondrial function during DOX treatment [180].

#### 3.1.2. Other Conventional Anti-Diabetic Treatments

Research further underscored pioglitazone’s potential in mitigating DOX-induced cardiomyopathy, though its cardioprotective effects remain incomplete. Animal studies suggested that pioglitazone administration attenuates DOX-induced left ventricular dysfunction, particularly during the acute and chronic phases, while offering limited protection in the hyperacute phase [181]. The cardiotoxicity associated with DOX was linked to elevated miR-130a levels and reduced PPAR-γ activity. Targeting miR-130a showed promise in mitigating cardiac damage by restoring PPAR-γ function in cardiomyocytes, thereby exerting anti-inflammatory and anti-apoptotic effects. Pioglitazone contributed to this protective mechanism by increasing PPAR-γ expression and decreasing miR-130a levels, although it failed to fully prevent DOX-induced cardiotoxicity [182,183].

Moreover, recent data underlined pioglitazone’s ability to reverse key biochemical markers associated with DOX-induced cardiomyopathy, such as altered thyroid hormone fractions and elevated troponin levels [184]. However, concerns persist regarding the potential exacerbation of cardiac injury via PPAR-γ agonism. Some reports suggested that PPAR-γ agonism may contribute to structural damage in heart tissue and alter critical biochemical markers, raising doubts about pioglitazone’s cardioprotective capacity [185]. While pioglitazone itself is not considered directly cardiotoxic, its activation of PPAR-γ could, under certain conditions, aggravate cardiac dysfunction. These findings indicate that the therapeutic use of pioglitazone in managing DOX-induced cardiomyopathy requires careful consideration of both its potential benefits and associated risks. Lastly, data on the role of SUs in chemotherapy-induced cardiotoxicity are lacking. However, one experimental study suggested that glibenclamide may exacerbate Adriamycin-promoted cardiotoxicity by activating oxidative stress-induced ER stress [186].

#### 3.1.3. Incretin Mimetics

Limited evidence also suggests that DPP-4 inhibitors may offer cardioprotective effects against DOX-induced cardiotoxicity. Similar to metformin, sitagliptin was shown to suppress pro-inflammatory cytokines, such as TNF-α, and inhibit NF-κB activation. Additionally, it mitigated oxidative damage by reducing lipid peroxidation and preserving anti-oxidant defenses in cardiac tissue. Sitagliptin also inhibited the upregulation of pro-apoptotic proteins [187]. Linagliptin also demonstrated comparable benefits, particularly in preserving myocardial fiber structure and reducing oxidative stress, evidenced by lower levels of malondialdehyde (MDA), a marker of lipid peroxidation, and preserved glutathione peroxidase (GPx) activity [188].

Preliminary research indicated that GLP-1 RAs may also have significant potential in mitigating DOX-associated cardiomyopathy. This potential was attributed to liraglutide’s capacity to reduce inflammation and apoptosis, mediated by the upregulation of AMPK activity, which may attenuate markers of cardiac injury and support cellular anti-oxidant systems [189]. However, conflicting data suggested that liraglutide may not effectively inhibit critical inflammatory mediators such as NF-κB. Furthermore, it appeared ineffective in restoring mitochondrial dynamics, as evidenced by unaltered levels of mitochondrial proteins, including protein optic atrophy-1 (OPA-1), mitofusin-2 (MFN-2), dynamin-related protein 1 (DRP-1), and topoisomerase 2β [190]. In contrast, recent evidence for semaglutide suggested cardioprotective qualities, with moderate to high dosages correlating with reductions in troponin and lactate dehydrogenase (LDH) levels, alongside improved histopathological outcomes [191]. Lastly, tirzepatide was shown to exhibit anti-inflammatory and anti-oxidant activities in the context of DOX-induced cardiomyopathy, likely through the activation of the PI3K/Akt signaling pathway [192].

#### 3.1.4. SGLT-2 Inhibitors

Among the various classes of anti-diabetic agents, SGLT-2 inhibitors have emerged as the most cardioprotective, making their potential to mitigate chemotherapy-related cardiotoxicity especially promising. A growing body of preclinical evidence notes this cardioprotective capacity, with distinct mechanisms of action identified across the major SGLT-2 inhibitors [193,194]. Notably, dapagliflozin was shown to exert pleiotropic benefits, largely through the activation of the PI3K/Akt signaling pathway. This activation led to a reduction in oxidative stress, suppression of inflammation, attenuation of cardiac hypertrophy, and mitigation of myocardial fibrosis. Furthermore, dapagliflozin showed efficacy in ameliorating mitochondrial dysfunction, alongside notable anti-arrhythmic properties, the latter attributed to its ability to mitigate DOX-induced sarcolemma injury and myocardial necrosis [195,196]. Empagliflozin likewise exhibited robust cardioprotective features, particularly in preventing adverse cardiac remodeling, even in the absence of DM [197]. Its additional anti-oxidant properties, potentially mediated via the inhibition of the JNK/STAT3 signaling cascade, further underscored its therapeutic promise in mitigating chemotherapy-induced cardiotoxicity [198]. Similar protective mechanisms also were observed with canagliflozin, which notably reduced cisplatin-associated cardiotoxic impacts [199]. Uniquely, canagliflozin extended its protective repertoire by mitigating carfilzomib-induced endothelial apoptosis through an AMPK-dependent pathway [200].

Beyond their anti-inflammatory and anti-oxidant properties, SGLT-2 inhibitors appeared to counteract chemotherapy-induced cardiotoxicity through a profound modulation of cellular energy metabolism. These agents activated key nutrient deprivation pathways, such as AMPK and sirtuins, while concurrently inhibiting nutrient excess signaling, including Akt/mTOR. This dual regulatory mechanism fostered enhanced autophagy, stimulated mitochondrial biogenesis via PGC-1α activation, and promoted ketogenesis, collectively supporting myocardial resilience under stress [201,202,203]. Supporting this mechanistic foundation, observational data corroborated the cardioprotective effects of SGLT-2 inhibitors in clinical settings. For example, a study by Gongora et al. involving 3033 diabetic cancer patients receiving anthracycline chemotherapy revealed a significant reduction in cardiac events (3% vs. 20%) and overall mortality (9% vs. 43%) in those treated with SGLT-2 inhibitors compared to controls [204]. Notably, recent research by Liu et al. suggested that empagliflozin may offer cardioprotective benefits in humans exposed to sorafenib by influencing pathways related to inflammation, fibrosis, DNA damage, and ferroptosis [205].

**Figure 4:**
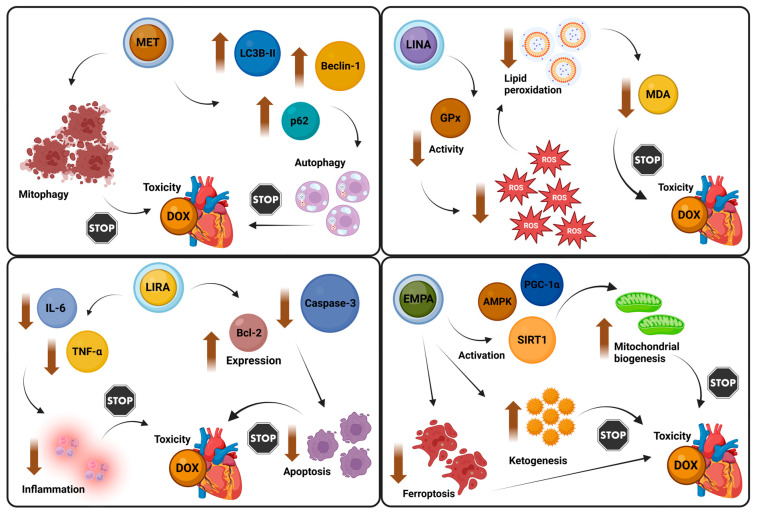
Schematic presentation of signaling pathways influenced by anti-diabetic drugs to counteract doxorubicin cardiotoxicity. Metformin may enhance cardiac function by modulating autophagy and mitophagy pathways, which is evidenced by the normalization of autophagy markers such as beclin-1, LC3B-II, and p62. Liraglutide demonstrates the ability to reduce inflammation and apoptosis by decreasing the levels of pro-inflammatory cytokines (IL-6 and TNF-α), downregulating the pro-apoptotic caspase-3, and upregulating the anti-apoptotic protein Bcl-2. On the other hand, linagliptin exhibits anti-oxidant properties, potentially reducing oxidative stress through the reduction of GPx activity, thereby limiting ROS generation, decreasing lipid peroxidation, and mitigating MDA formation. This process preserves cellular integrity and improves myocardial fiber structure, which is crucial in alleviating DOX-induced cardiomyopathy. Lastly, empagliflozin is illustrated to potentially mitigate DOX-related cardiac injury by enhancing mitochondrial biogenesis through the activation of the AMPK/SIRT1/PGC-1α pathway. Additionally, it may reduce ferroptosis and enhance ketogenesis, contributing further to its cardioprotective effects [176,188,189,199,200,201]. Abbreviations: AMPK: AMP-activated protein kinase; Bcl-2: B cell lymphoma 2; DOX: Doxorubicin; EMPA: Empagliflozin; GPx: Glutathione peroxidase; IL-6: Interleukin 6; LC3B-II: Microtubule-associated protein 1A/1B light chain 3B, form II; LINA: Linagliptin; LIRA: Liraglutide: MDA: Malondialdehyde; MTF: Metformin; PGC-1α: Peroxisome proliferator-activated receptor gamma coactivator 1-alpha; ROS: Reactive oxygen species; SIRT1: Sirtuin 1; TNF-α: Tumor necrosis factor-alpha. Created with www.BioRender.com.

In light of these findings, it is evident that anti-diabetic therapies, particularly metformin and SGLT-2 inhibitors, may offer substantial cardioprotective gains for oncology patients undergoing DOX treatment. This is unsurprising, as inflammation, oxidative stress, apoptosis, and mitochondrial dysfunction, key drivers of DOX-induced cardiomyopathy [206], are pathways that anti-diabetic agents were shown to modulate in experimental models. Moving forward, well-designed randomized clinical trials are essential to confirm these cardioprotective impacts and determine whether glucose-lowering treatments can be effectively repurposed to prevent chemotherapy-induced cardiotoxicity. Figure 4 illustrates the key molecular mechanisms by which various anti-diabetic agents mitigate DOX-induced cardiotoxicity through the modulation of distinct signaling pathways.

### 3.2. Anti-Diabetic Agents as Modulators of Chemotherapy-Induced Toxicities: Beyond Cardiotoxicity

Beyond their cardioprotective benefits, anti-diabetic therapies also play a vital role in mitigating other chemotherapy-related toxicities, particularly those affecting the kidneys and liver. These protective effects are driven by shared mechanisms, including the modulation of inflammation, oxidative stress, apoptosis, and mitochondrial dysfunction, processes that contribute to both cardiac and non-cardiac toxicities. Anti-diabetic agents have shown significant promise in reducing chemotherapy-induced acute kidney injury (AKI), especially in cases involving cisplatin. Among these, metformin emerged as a key protective agent, known for its ability to activate AMPK signaling and enhance autophagic flux in renal tubular cells, mechanisms that reduce apoptosis and preserve renal function [207]. Similarly, gliclazide and pioglitazone, which act through anti-oxidant and anti-inflammatory pathways, also demonstrated potent nephroprotective properties [208,209]. Additionally, DPP-4 inhibitors showed promise in preclinical models, mitigating renal damage by modulating key inflammatory and fibrotic pathways, such as the MAPK/JNK signaling pathway and the NLRP3 inflammasome [210,211]. Furthermore, GLP-1 RAs, such as liraglutide, particularly in combination with curcumin, exhibited nephroprotective effects by enhancing the Nrf2/HO-1 pathway and regulating GSK-3β activity [212]. Moreover, SGLT-2 inhibitors, including dapagliflozin and empagliflozin, offer renal protection by reducing oxidative stress, renal fibrosis, and apoptosis, particularly in cases of doxorubicin-induced nephrotoxicity [213,214].

Anti-diabetic treatments also showed promise in managing chemotherapy-related liver injury. Metformin lowered doxorubicin accumulation in the liver via the inhibition of organic cation transporters [215], while gliclazide and pioglitazone provided hepatoprotection against cisplatin and tamoxifen-induced liver injury by modulating oxidative stress and inflammation [216,217]. SGLT-2 inhibitors further shielded liver tissue by preserving structural integrity and reducing oxidative stress, with dapagliflozin showing enhanced efficacy in combination with silymarin [218]. Glucose-lowering treatments further showed protective effects in various organ systems beyond the kidneys and liver. Metformin reduced cisplatin-induced ototoxicity by promoting SIRT3 expression and restoring glucose uptake in auditory cells [219]. In chemotherapy-induced peripheral neuropathy, DPP-4 inhibitors like alogliptin, along with pioglitazone, alleviated neurotoxicity through anti-inflammatory and anti-oxidant actions [220,221]. Pioglitazone also reduced cisplatin-induced testicular damage by downregulating the toll-like receptor 4/myeloid differentiation factor 88 (TLR4/MyD88)/NF-κB signaling pathway [222], while liraglutide improved reproductive function following doxorubicin exposure [223]. Additionally, metformin was shown to reduce cisplatin-induced genotoxicity in bone marrow cells by decreasing micronucleated erythrocytes and oxidative stress, while also improving hematological profiles [224].

While preclinical data strongly support the protective outcomes of anti-diabetic agents against chemotherapy-induced toxicities, clinical translation remains in its early stages, though initial findings are promising. A randomized controlled trial investigating metformin in non-DM breast cancer patients receiving Adriamycin-cyclophosphamide plus paclitaxel demonstrated a significant reduction in peripheral neuropathy, oral mucositis, and fatigue. Additionally, metformin preserved cardiac function and reduced the risk of developing fatty liver, suggesting its broad potential in mitigating multiple chemotherapy-related toxicities [225]. Further clinical evidence highlighted metformin’s efficacy in reducing paclitaxel-induced peripheral neuropathy, improving treatment tolerance, and potentially enhancing long-term outcomes [226].

In DM cancer patients, the nephroprotective results of DPP-4 inhibitors also were demonstrated. A study by Iwakura et al. found that DPP-4 inhibitor users experienced a significantly lower decline in estimated glomerular filtration rate (eGFR) two weeks after cisplatin treatment and a reduced incidence of AKI compared to non-users (25% vs. 64%) [227]. These early clinical findings suggest that anti-diabetic drugs may offer significant adjunctive benefits in reducing chemotherapy-induced toxicities. However, further clinical trials are necessary to validate their efficacy and expand their use in the oncological settings.

### 3.3. Decoding the Impact of Anti-Diabetic Therapies on Chemotherapy Outcomes Beyond Toxicity Mitigation

#### 3.3.1. Traditional Anti-Diabetic Treatments

Metformin showed promising results in both preclinical and clinical studies across various malignancies, notably in gynecological cancers [228,229]. Of special interest is the synergistic effect of metformin with doxorubicin in breast cancer, where it appears to facilitate the selective eradication of cancer stem cells, significantly enhancing the therapeutic impact of chemotherapy [230]. In ovarian cancer, metformin, when combined with carboplatin, demonstrated adjunctive effects by inhibiting the Akt/mTOR signaling pathway [231]. Notably, metformin may also overcome cisplatin resistance in ovarian cancer cells by inducing autophagy [232]. Data from non-DM breast cancer patients (stages II and III) showed that the combination of metformin with neoadjuvant chemotherapy resulted in a lower residual cancer burden score (40.7% vs. 68.8% in class 3 patients) [233].

Metformin’s potential was further explored in CRC, where combination therapy with 5-FU showed tumor size reduction through the modulation of NF-κB signaling [234]. There is also compelling evidence suggesting that metformin may enhance the chemosensitivity of CRC cells to the oxaliplatin-based regimen (FuOx). In vitro studies demonstrated that metformin significantly improved FuOx’s efficacy by inhibiting cell proliferation, colony formation, and migration, while also inducing cell cycle arrest and promoting late apoptosis through the regulation of mitochondrial proteins. These findings were further corroborated by in vivo experiments, where the combination of metformin and FuOx resulted in a significantly greater reduction in tumor volume compared to either treatment alone [235]. However, metformin’s efficacy appeared limited in advanced pancreatic cancer, where its addition to gemcitabine and erlotinib regimens did not improve patient outcomes [236].

Preliminary and clinical data also suggested a role for metformin in hematological malignancies. For instance, in acute myeloid leukemia, metformin was shown to enhance the anti-tumor effect of a co-administration with cytarabine by inhibiting the mTOR complex1 (mTORC1/p70S6) kinase signaling pathway [237]. Similarly, in lymphoma, the addition of metformin to a regimen of rituximab and anthracyclines significantly improved both progression-free and overall survivals, compared to other anti-hyperglycemic agents. This study further demonstrated that metformin could potentiate the anti-diffuse large B cell lymphoma (DLBCL) effects of DOX and rituximab [238].

Synergistic results of pioglitazone were also reported, particularly in the context of breast cancer treatment. In combination with cisplatin, pioglitazone showed anti-apoptotic features, offering a promising adjunct therapy for women with TNBP. Compared to cisplatin monotherapy, co-administration with pioglitazone significantly enhanced tumor cell apoptosis in a dose-dependent manner, as indicated by reduced expression of the anti-apoptotic protein Bcl-2 and increased expression of pro-apoptotic proteins such as cleaved-poly-ADP ribose polymerase (PARP) and caspase-9 [239].

A significant body of research further explored the role of DPP-4 inhibition in chemotherapy outcomes, yielding mixed findings [86,240]. Several endogenous DPP-4 substrates, such as CXCL12, human granulocyte-macrophage colony-stimulating factor (GM-CSF), and IL-3, may have diverse consequences on chemotherapy-induced damage to healthy tissues [241]. For instance, certain studies suggested that DPP-4 inhibition may contribute to breast cancer chemoresistance by increasing levels of CXCL12, leading to the induction of EMT in tumors [242]. However, other findings highlight the potential of targeting DPP-4 to inhibit angiogenesis in 5-FU-resistant colon cancer. In this context, exosomal DPP-4 was proposed as a promising prognostic marker [243].

#### 3.3.2. Novel Anti-Diabetic Therapies

Similar favorable outcomes in chemotherapy efficacy were observed with exenatide, which demonstrated the potential to alleviate resistance to enzalutamide in advanced prostate cancer. In a murine experimental model, the combination of exenatide and enzalutamide significantly suppressed tumor growth compared to enzalutamide monotherapy. This combinatorial treatment not only mitigated the enzalutamide-induced invasion and migration of prostate cancer cells but also reduced the levels of Akt and mTOR, which were activated in response to enzalutamide. Importantly, the dual therapy resulted in a further decrease in nuclear androgen receptor localization, despite exenatide alone exerting no effect on nuclear androgen receptor levels [244].

Recent research also underlined the potential of combining SGLT-2 inhibitors with cisplatin therapy as a promising strategy to overcome cisplatin resistance in hepatoblastoma. Specifically, dapagliflozin, through SGLT-2 inhibition, reduced glucose uptake, a critical factor in the development of cisplatin resistance [245]. Similarly, canagliflozin showed therapeutic promise in HCC by inhibiting cell proliferation and migration through mechanisms that extended beyond its primary SGLT-2 target. Notably, canagliflozin induced the downregulation of pyruvate kinase M2 (PKM2), leading to c-Myc degradation, which disrupted glutamine metabolism and induced glutamine starvation. This metabolic alteration promoted ferroptosis, sensitizing cancer cells to cisplatin [246].

Furthermore, the combination of dapagliflozin and canagliflozin was reported to enhance the anti-cancer efficacy of paclitaxel in ovarian cancer and oral squamous cell carcinoma. Mechanistic insights from studies on ovarian cancer cells indicated that canagliflozin augmented paclitaxel-induced apoptosis and DNA damage while impairing the spindle assembly checkpoint (SAC) through the downregulation of cyclin B1 and phosphorylated BUB1 mitotic checkpoint kinase 1 (BUBR1). This ultimately accelerated premature mitotic exit and contributed to the accumulation of aneuploid cells [247]. Recently, Karim et al. investigated the synergistic effects of prominent SGLT-2 inhibitors, canagliflozin, dapagliflozin, and empagliflozin, when used in conjunction with doxorubicin to address chemotherapy resistance. Their findings demonstrated that the combination of canagliflozin and doxorubicin significantly enhanced cytotoxicity in breast cancer cells, attributed to a reduction in glucose consumption and lower levels of intracellular ATP and lactate, both of which are essential for cancer cell proliferation [248]. While emerging preclinical studies support the potential of SGLT-2 inhibitors to enhance chemotherapy efficacy across various cancers, current clinical data remain limited. Notably, a case report showed that the combination of SGLT-2 inhibitors with cetuximab can reduce tumor size and carcinoembryonic antigen levels in metastatic CRC, warranting further investigation into these combinations in clinical settings [249].

Overall, the aforementioned findings highlight the promising role of anti-diabetic drugs in overcoming drug resistance and improving therapeutic outcomes. However, to substantiate these observations and explore their broader applicability, comprehensive future clinical studies are essential.

## 4. Unleashing the Benefits of Immunotherapy with Anti-Diabetic Treatment: Emerging Preclinical Insights with Potential Future Clinical Applications

Beyond their role in improving chemotherapeutic outcomes, anti-diabetic drugs are increasingly recognized for their potential to enhance the efficacy of immunotherapies. The beneficial impact of anti-diabetic medications in this context is likely due to their ability to reprogram the TME, promoting a cellular milieu less conducive to tumor progression. Current research is exploring how these agents may emulate or augment the effects of immune checkpoint inhibitors (ICIs), such as programmed cell death protein-1 (PD-1) and programmed death-ligand-1 (PD-L1) inhibitors, with emerging evidence suggesting that they may also interact synergistically with anti-cytotoxic T lymphocyte-associated protein-4 (CTLA-4) therapies.

### 4.1. Metformin

Metformin has emerged as a powerful adjunct in enhancing the efficacy of ICI therapies, particularly those targeting the PD-1 pathway. This enhancement is primarily achieved through metformin’s modulation of the TME, by promoting the infiltration of CD4^+^ T cells essential for anti-tumor responses, while simultaneously diminishing the population of regulatory T cells (Tregs) that typically exert immunosuppressive effects. Through the upregulation of IL-17A, metformin reinforces immune activation, and it also induces epigenetic modifications in Treg DNA to curtail their suppressive capabilities. Additionally, metformin activates AMPK, initiating a signaling cascade involving SIRT2 that leads to the downregulation of CCR8 on Tregs. This attenuation of CCR8 expression diminishes immune evasion, thereby enhancing anti-tumor immunity and augmenting the therapeutic efficacy of anti-PD-1 therapies [250]. In addition to its effects on the TME, metformin influences immune checkpoint gene expression across several malignancies, including breast cancer and CRC [251]. Specifically, it was shown to reduce PD-L1 expression through various mechanisms, including PD-L1 degradation in the ER and activation of the Hippo signaling pathway [252,253]. By downregulating PD-L1, metformin alleviates the inhibitory signaling that typically constrains cytotoxic T lymphocyte activity, thereby enhancing the potential for synergistic effects with CTLA-4 inhibitors [252].

In recent years, studies emphasized the important relationship between metformin and the gut microbiome, suggesting that these interactions may enhance the effectiveness of ICI therapies [253]. While the precise mechanisms are still under investigation, metformin’s high concentrations in the intestines relative to its systemic levels indicate that it plays a crucial role in shaping gut microbiota [254]. The drug promotes a balanced composition of gut microbiota, facilitating immune regulation through various mechanisms, including the influence of bile acid dehydroxylation and the activation of intestinal Farnesoid X receptor (FXR) signaling, which collectively help mitigate metabolic dysregulation [255]. Additionally, metabolites derived from the gut, such as short-chain fatty acids (SCFAs) and bile acids, contribute significantly to immune regulation. This suggests that metformin’s ability to modulate the microbiome may create a supportive environment for improved anti-tumor immunity when used alongside ICIs [256]. However, inter-individual variability in the concentrations of metformin within the gut and the composition of the microbiome complicate the reliable translation of these microbiota-mediated benefits into clinical practice [251].

Emerging clinical evidence notes metformin’s potential to enhance survival outcomes in patients undergoing ICI therapy. A retrospective study in Taiwan involving 878 diabetic patients demonstrated significantly prolonged overall survival (OS) and progression-free survival (PFS) among metformin users compared to non-users (OS: 15.4 vs. 6.1 months; PFS: 5.1 vs. 1.9 months), even after adjusting for confounding factors such as age and cancer stage [257]. Similarly, in a multicenter cohort of 516 patients with solid tumors treated with ICIs, metformin showed specific survival benefits in the lung cancer subgroup, correlating with improved OS and PFS [258]. Furthermore, bioinformatic analyses identified five metformin-targeted genes in NSCLC, all downregulated in cancerous tissue and associated with an increased infiltration of immune cells, including CD4^+^ and CD8^+^ T cells [251]. While metformin’s role as an adjunct in immunotherapy is increasingly recognized, further investigation is required to fully elucidate its mechanisms and optimize its clinical application.

### 4.2. Other Conventional Anti-Diabetic Therapies

In a similar vein, pioglitazone showed significant potential in enhancing immunotherapy efficacy, particularly in CRC. Pioglitazone promoted PD-L1 degradation, enhancing immune-mediated tumor recognition. Jia et al. demonstrated that pioglitazone reduced PD-L1 protein levels in cancer cells through autophagy, independent of changes in gene expression. This was achieved via the activation of PPAR-γ, which facilitated PD-L1 degradation by promoting its translocation to the lysosome, thereby increasing the interaction between PPAR-γ and PD-L1. When combined with PD-1 inhibitors, pioglitazone enhanced treatment efficacy by reducing PD-L1 levels and promoting the infiltration of cytotoxic CD8^+^ T cells, thereby amplifying the anti-tumor immune response in CRC [259]. On the other hand, the impact of SUs on immunotherapy outcomes is less well documented, although recent research suggests that glibenclamide may enhance the therapeutic efficacy of PD-1 blockade. Specifically, glibenclamide acted as an agonist of the transketolase 1 gene (TKTL1), which plays a crucial role in mediating the immune response to PD-1 therapy in kidney clear cell carcinoma. By increasing TKTL1 expression, glibenclamide promoted immune cell recruitment within the TME, potentially augmenting the effectiveness of anti-PD-1 treatment [260].

DDP-4 inhibitors further showed promising potential in modulating immune responses and improving therapeutic outcomes for cancer patients who exhibited resistance to conventional PD-L1 treatments. Recent evidence suggests that combining anagliptin with PD-L1 antibodies led to significantly improved outcomes in NSCLC compared to PD-L1 blockade alone. This enhancement was primarily due to anagliptin’s ability to inhibit macrophage formation and prevent M2 polarization within the TME, achieved through the reduction in ROS production and the suppression of signaling pathways that promote monocyte-to-macrophage differentiation [261]. A recent investigation evaluated the effects of six commonly prescribed anti-diabetic medications on the efficacy of anti-PD-1 immunotherapy in syngeneic mouse models of CRC and melanoma, revealing differential impacts on tumor suppression. Notably, sitagliptin was found to enhance the efficacy of anti-PD-1 therapy, whereas metformin exhibited a neutral effect. In contrast, glimepiride, pioglitazone, and insulin were associated with decreased tumor inhibition [262].

### 4.3. Novel Anti-Diabetic Treatments

#### 4.3.1. GLP-1 and Dual GIP/GLP-1 Receptor Agonists

Although direct data on the impact of GLP-1 RAs on ICI therapy are lacking, emerging evidence suggests potential benefits. Preliminary findings indicated that liraglutide may enhance the anti-tumor efficacy of PD-1 inhibitors by targeting neutrophil extracellular traps (NETs) [263]. NETs, composed of DNA, histones, and proteins, are released by neutrophils in response to tissue damage and contribute to cancer progression by creating a pro-inflammatory environment that fosters tumor growth, metastasis, and immune evasion [264]. Chen et al. demonstrated that liraglutide significantly reduced NET markers, including myeloperoxidase (MPO), double-stranded DNA (dsDNA), and elastase, in lung and liver cancer models, while inhibiting NET formation by reducing ROS. When combined with PD-1 inhibitors, liraglutide facilitated sustained CD8^+^ T cell responses, thereby offering protection against tumor recurrence [263].

The paradoxical relationship between obesity and improved outcomes in certain cancers treated with ICIs, such as melanoma and RCC, presents a complex and evolving landscape. Although the underlying mechanisms are not fully understood, obesity appears to influence several key factors, including inflammation, cancer cell metabolism, and angiogenesis [265]. Human tissue research indicated that obesity is associated with reductions in OXPHOS, creating a state of metabolic rest that may enhance the effectiveness of cancer therapies [266]. In preclinical models of RCC, obesity was linked to the decreased expression of fatty acid synthase, a critical enzyme in lipid metabolism that supports cancer cell survival and proliferation. Additionally, obesity seemed to increase immune cell infiltration and hypoxia in tumor-adjacent adipose tissue, potentially boosting the immune response [267]. Interestingly, clinical data from patients with stage 4 cancer treated with anti-PD-1/PD-L1 inhibitors showed that overweight or obese patients often experienced more favorable outcomes, such as prolonged time to treatment failure, compared to those with a normal body mass index (BMI) [268].

This phenomenon, often referred to as the “obesity paradox”, highlights the potential benefits of excess weight in enhancing the immune response during ICI therapy [265]. Thus, this paradox raises important questions about the impact of weight loss induced by GLP-1 analogs and GIP/GLP-1 agonists, which are commonly prescribed for their weight-reducing effects. It remains crucial to investigate whether weight loss from these therapies could inadvertently reduce the therapeutic benefits observed in obese patients undergoing immunotherapy for certain cancer types.

#### 4.3.2. SGLT-2 Inhibitors

Recent experimental studies revealed the critical role of SGLT-2 in regulating immune checkpoint activities within cancer cells, suggesting that SGLT-2 inhibitors may serve as effective modulators of anti-tumor immunity. The immunomodulatory properties of these drugs were demonstrated across various agents, with canagliflozin emerging as a prominent example. Canagliflozin exhibited a dual role in both glycemic control and immunoregulation by impeding T cell activation, proliferation, and effector functions through the inhibition of key signaling pathways, such as ERK and mTORC1. This inhibition reduced c-Myc levels, a transcription factor essential for metabolic processes critical to T cell function [269]. In cancer therapy, canagliflozin’s favorable immunomodulatory effects were noted through its interaction with PD-L1. By disrupting the SGLT-2/PD-L1 axis, canagliflozin promoted the degradation of PD-L1 via the ubiquitin-proteasome pathway, thereby enhancing T cell-mediated cytotoxicity. This action limited tumor progression and improved the efficacy of immune checkpoint blockade therapies, akin to the effects observed with PD-1 monoclonal antibodies [270].

Nevertheless, SGLT-2 silencing extended to other immune checkpoints, such as CTLA-4. Dapagliflozin and empagliflozin were shown to mitigate the adverse effects associated with ipilimumab, primarily by modulating key inflammatory mediators [271,272]. In hyperglycemic states, empagliflozin not only reduced ROS formation but also decreased the production of cytokines and growth factors, including IL-6, TGF-β, VEGF, and leukotrienes. This reduction led to a downregulation of NF-κB and NLRP3 inflammasome activity [272]. Importantly, clinical data suggested that patients receiving ICIs who concurrently were using SGLT-2 inhibitors experienced a significant reduction in all-cause mortality compared to non-users (21% vs. 59%) over a long-term follow-up of nearly two years. This survival benefit persisted despite no significant impact on major adverse cardiovascular events (MACE) [273].

**Figure 5:**
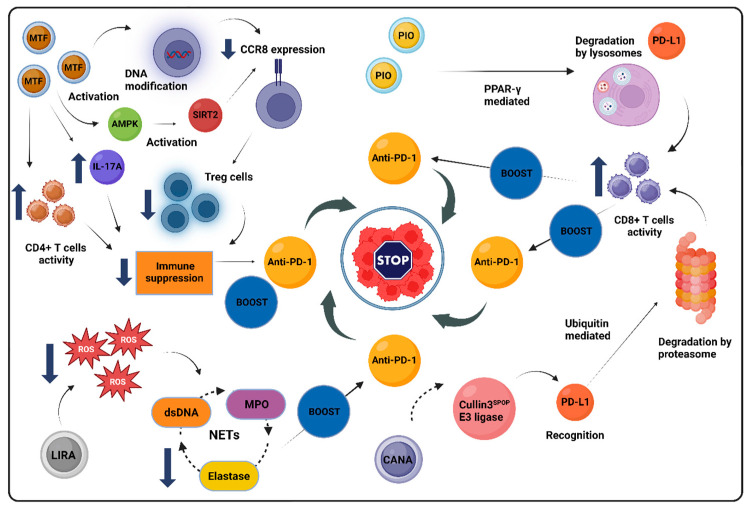
Modulation of immune responses by anti-diabetic drugs in bolstering anti-PD-1 immunotherapy outcomes. Metformin enhances the infiltration of CD4^+^ T cells, crucial for initiating and sustaining anti-tumor immunity, while concurrently reducing the population of Tregs, which suppress immune responses within the TME. Moreover, metformin increases IL-17A levels and induces epigenetic modifications in Tregs, resulting in reductions in their immunosuppressive capabilities. By activating AMPK and triggering a downstream signaling cascade involving SIRT2, metformin promotes the downregulation of CCR8 on tumor-infiltrating Tregs. This downregulation decreases immune evasion and boosts anti-tumor responses, particularly when combined with PD-1 inhibitors. Pioglitazone and canagliflozin also contribute to improved ICI efficacy by promoting the degradation of PD-L1, a checkpoint protein that inhibits T cell activity. Pioglitazone acts as a PPAR-γ agonist, facilitating PD-L1 localization to lysosomes, while canagliflozin enhances PD-L1 recognition by the Cullin3SPOP E3 ligase for ubiquitination and proteasomal degradation. This degradation leads to increased infiltration of cytotoxic CD8^+^ T cells, thereby reinforcing the anti-tumor immune response. Additionally, liraglutide, through its GLP-1 receptor agonism, mitigates oxidative stress, resulting in reduced production of NETs, which can promote inflammation and tumor progression. By decreasing NET levels, liraglutide aids in alleviating tumor-promoting inflammation, thereby further enhancing the efficacy of ICIs [250,259,263,270]. Abbreviations: AMPK: AMP-activated protein kinase; Anti-PD-1: Anti-programmed cell death protein 1; CANA: Canagliflozin; CCR8: C-C chemokine receptor 8; CD4^+^ T cells: Cluster of differentiation 4 positive T cells; CD8^+^ T cells: Cluster of differentiation 8 positive T cells; dsDNA: Double-stranded deoxyribonucleic acid; IL-17A: Interleukin-17A; ICI: Immune checkpoint inhibitor PD-L1: Programmed death-ligand 1; LIRA: Liraglutide; MPO: Myeloperoxidase; MTF: Metformin; NETs: Neutrophil extracellular traps; PIO: Pioglitazone; PPAR-γ: Peroxisome proliferator-activated receptor gamma; ROS: Reactive oxygen species; SIRT2: Sirtuin 2; TME: Tumor microenvironment; Tregs: Regulatory T cells. Created with www.BioRender.com.

These findings suggest that incorporating SGLT-2 inhibitors into cancer immunotherapy regimens holds great potential for enhancing patient outcomes by leveraging their immunomodulatory properties. However, to fully harness this potential, future clinical studies are necessary to elucidate the precise mechanisms driving these effects and to optimize treatment strategies for patients undergoing ICI therapy. Figure 5 illustrates the mechanisms by which anti-diabetic drugs may modulate immune responses, contributing to enhanced anti-PD-1 therapeutic outcomes.

## 5. Translating Preclinical Insights into Clinical Cancer Outcomes: Can We Draw Definitive Conclusions?

### 5.1. Metformin

In recent years, extensive clinical research explored metformin’s potential role in influencing cancer risk and progression. These investigations addressed both the overall incidence of malignancies and specific cancer types, yielding heterogeneous findings. A particular focus was placed on metformin’s association with breast cancer outcomes. While certain studies suggested a potential reduction in cancer incidence and mortality relative to other anti-diabetic therapies, others found no statistically significant association between metformin use and cancer prognosis [274]. Importantly, studies indicating a favorable effect of metformin often emphasized its time- and dose-dependent influence. Moreover, the post-diagnosis administration of metformin was correlated with lower mortality rates across various cancers, including breast, lung, and endometrial malignancies. Interestingly, long-term metformin administration, specifically over a period exceeding five years, was linked to a diminished risk of brain tumors [275,276].

Metformin’s potential therapeutic benefits extended to gastrointestinal malignancies, with evidence suggesting a positive effect on HCC, pancreatic cancer, and CRC. For example, a recent study by Tarhini et al. reported enhanced OS and disease-free survival in patients with T2DM and CRC who received metformin therapy. However, it is crucial to consider that patients in the metformin cohort were generally younger and exhibited higher body mass indices compared to non-users, which may have confounded the outcomes [277]. Conversely, data from the Korean National Health Insurance Service-National Health Screening Cohort indicated an increased risk of pancreatic cancer among diabetic women treated with metformin, while no significant risk difference was observed between metformin users and non-users among diabetic men [278].

The impact of metformin on urogenital cancers remains ambiguous. Some evidence suggests a potential reduction in the incidence of kidney cancer with metformin use, though its effects on prostate cancer are more nuanced [279]. While metformin did not appear to elevate the overall risk of prostate cancer, it was associated with a 14% increase in the incidence of low-grade prostate cancer and a 25% reduction in the risk of high-grade disease [280]. Similarly, research on bladder, ovarian, and endometrial cancers yielded divergent results, with studies reporting positive, neutral, or even contradictory outcomes [281,282,283,284,285].

Meta-analyses provided further insights into the relationship between metformin and cancer, though inconsistencies persist. A meta-analysis conducted by O’Connor et al., which synthesized data from 166 studies on cancer incidence, suggested that metformin use was associated with a reduced overall cancer risk, particularly in gastrointestinal, urinary tract, and hematologic malignancies. Additionally, some studies supported a positive association between metformin use and a reduced risk of multiple myeloma in DM individuals [286,287]. However, a more recent meta-analysis by Mesquita et al. contradicted these findings, reporting no significant anti-cancer effects of metformin in T2DM patients, regardless of body weight or prediabetic status [288]. Although certain meta-analyses suggested metformin may not be effective in preventing certain cancers, such as endometrial cancer, the drug may confer secondary benefits, including a reduced mortality risk and prolonged progression-free survival [289].

### 5.2. Insulin Therapy and Sulfonylureas

The association among insulin, oral insulin secretagogues, and cancer risk was as a significant focus of research, particularly in the preceding years. As previously discussed, hyperinsulinemia may act as a facilitator in cancer development, prompting numerous clinical studies to investigate whether therapies that elevate insulin levels contribute to tumorigenesis in DM patients. A pivotal study by Vicentini et al. identified a 20% increase in cancer incidence, particularly among T2DM subjects undergoing insulin therapy, when compared to non-DM individuals. This increased incidence was especially pronounced for liver, pancreatic, and bladder cancers. Notably, T1DM patients also exhibited a heightened risk of bladder cancer, suggesting that the oncogenic potential of insulin therapy may extend beyond T2DM populations [290].

Further research examined the cancer risks associated with different insulin secretagogues. Data from Taiwan’s National Health Insurance database, which encompassed over 8000 cancer cases in T2DM patients, demonstrated that insulin use was linked to the highest overall cancer risk, followed by glinides and SUs. Notably, while first- and second-generation SUs were associated with a moderate increase in cancer risk, third-generation agents such as glimepiride did not show a significant association with cancer. The observed variability in risk profiles underscores the differential oncogenic potential of specific agents, with liver cancer being particularly associated with SU use [291].

Comparative analyses between insulin secretagogues and metformin consistently revealed a more favorable cancer risk profile for the latter. A population-based cohort study tracking over 10,000 patients reported higher cancer-related mortality among SU and insulin users compared to those receiving metformin. During a 5.4-year follow-up period, cancer-related mortality was 4.9% among SU users, 5.8% among insulin users, and 3.5% among metformin users, suggesting that metformin may offer protective effects against cancer, while insulin-promoting therapies may elevate oncogenic risk. However, the precise mechanisms underlying these observations remain unclear, leaving it uncertain whether the adverse outcomes were primarily attributable to insulin-promoting therapies or whether metformin’s protective properties were responsible [292].

Despite growing evidence, the relationship between SUs and cancer risk remains inconclusive. For instance, a systematic review by Chen et al., encompassing data from 77 clinical studies, did not confirm a definitive association between SU use and increased cancer risk. Moreover, randomized controlled trials (RCTs) did not consistently demonstrate significant oncogenic risks linked to SUs, and in some instances, these agents were even associated with protective effects against certain cancers, such as prostate cancer, particularly in low-risk populations. The inconsistency in findings may be due to variations in study design, patient demographics, and the pharmacological characteristics of different SUs [293]. For example, gliclazide, which stimulates insulin release in response to meals, may result in lower overall insulin exposure compared to glibenclamide, which maintains prolonged systemic insulin levels. Since hyperinsulinemia is a key driver of tumor growth, agents like gliclazide that limit sustained insulin exposure may carry a lower oncogenic risk than those that promote persistent hyperinsulinemia [56,294].

### 5.3. Pioglitazone

Numerous clinical studies and meta-analyses, including real-world data, implicated pioglitazone in a possible increased risk of bladder cancer, with this risk appearing to be dose- and time-dependent. Variability across geographical regions and potential influence from study funding sources further complicated these findings [295,296,297]. Early concerns were raised by the PROactive trial, a pivotal study on pioglitazone, which reported a higher incidence of bladder cancer in the pioglitazone group compared to the placebo group, although no significant difference was observed in the overall incidence of malignancies [298]. Importantly, this increased risk seemed to be drug specific, as rosiglitazone, another thiazolidinedione, was not significantly associated with bladder cancer [299].

However, emerging studies cast doubt on the validity of this association, with some reporting no significant link between pioglitazone use and bladder carcinogenesis, even in ethnically diverse populations [300,301]. For instance, a recent case-control study involving 6440 Asian-Indian patients with T2DM found no increased incidence of bladder cancer among pioglitazone users. Despite these findings, the study’s authors recommended caution, particularly in patients with a history of hematuria, and identified age over 58 as a significant risk factor for bladder cancer [300]. In light of the ongoing debate, the U.S. Food and Drug Administration (FDA) continues to advise against pioglitazone use in individuals with a history of hematuria or bladder cancer. Conflicting evidence also exists regarding pioglitazone’s association with other urinary tract cancers, such as prostate cancer, where some studies indicated a possible increased risk, while renal cancer showed neutral findings [302,303,304].

Pioglitazone’s role in other malignancies was also investigated, though the results remain inconclusive. Research on breast and lung cancers produced mixed findings, with some studies suggesting potential therapeutic benefits, while others report no significant association [302,305]. Pioglitazone was evaluated for its potential role in lung cancer chemoprevention, especially among high-risk current and former smokers. While a trend towards decreased Ki-67 labeling in former smokers with baseline dysplasia treated with pioglitazone was observed, no overall improvement in endobronchial histology was detected, though histological improvements were noted in certain lesions [306].

Moreover, pioglitazone may have a protective effect against certain gastrointestinal cancers, particularly HCC, likely due to its positive impact on liver steatosis and fibrosis [307]. Some evidence suggests that prolonged pioglitazone use may be inversely correlated with HCC risk, especially in T2DM subjects with predisposing risk factors [308]. Meta-analyses similarly point to a potential protective role for pioglitazone in CRC [309]. While PPAR-γ agonism may reduce the risk of some cancers, such as breast and prostate cancers, its relationship with pancreatic cancer remains unclear. Although some studies suggested a heightened risk, a recent meta-analysis found no significant association. By contrast, insulin and SUs demonstrated a stronger association with increased pancreatic cancer risk in DM patients, with variations noted across ethnic populations [310,311].

### 5.4. Incretin Mimetics

#### 5.4.1. DDP-4 Inhibitors

DPP-4 inhibitors were a subject of considerable debate, particularly regarding their potential association with thyroid and pancreatic cancers. While preclinical studies raised concerns about a possible link between DPP-4 inhibition and increased cancer risk, clinical meta-analyses did not substantiate these findings. Specifically, no statistically significant increase in thyroid cancer incidence was observed among patients treated with DPP-4 inhibitors, despite experimental data suggesting a potential risk [312]. Similarly, the association between DPP-4 inhibitors and pancreatic cancer remains inconclusive. Although concerns were raised, clinical studies largely failed to demonstrate a clear, consistent elevation in pancreatic cancer incidence among patients using these drugs [313,314].

On a more positive note, some clinical data indicated the potential benefits of DPP-4 silencing for certain cancer types, particularly CRC cancer. Diabetic patients treated with DPP-4 inhibitors, such as sitagliptin, showed improved disease-free survival rates and lower recurrence rates for CRC compared to those treated with other anti-diabetic agents, including metformin [315]. In addition to CRC, evidence points to a potential protective effect of DPP-4 inhibitors against melanoma. Compared to SUs, DPP-4 inhibitors were associated with a 23% reduction in melanoma risk, although no significant difference was observed regarding non-melanoma skin cancers [316].

However, not all studies support favorable outcomes across cancer types. For instance, investigations into breast cancer did not demonstrate significant survival advantages in patients treated with DPP-4 inhibitors [317]. Similarly, a retrospective analysis of metastatic RCC found no substantial clinical benefit from DPP-4 inhibition, further highlighting the variability in cancer-related outcomes associated with these drugs [318].

#### 5.4.2. GLP-1 RAs

Through the last years, concerns emerged regarding the potential link between GLP-1RAs and the development of thyroid cancer, particularly MTC [319]. Despite these worries, several RCTs failed to demonstrate significant elevations in calcitonin levels or an increased risk of MTC among patients treated with GLP-1RAs [320,321]. However, retrospective observational studies and pharmacovigilance reports suggested a possible association between GLP-1RA use and thyroid malignancies, which complicated the interpretation of clinical safety data [322,323]. As previously mentioned, preclinical studies showed that GLP-1 receptors were expressed in thyroid C-cells and that GLP-1 receptor agonism can promote C-cell hyperplasia and tumorigenesis in rodent models. Importantly, these effects appeared to be less pronounced in humans [98,99,100]. A recent meta-analysis encompassing 64 clinical trials, 26 of which included thyroid cancer cases, identified a moderate relative risk increase for thyroid cancer associated with GLP-1RA treatment. Nevertheless, the absolute risk increment was small, and no statistically significant correlation was found for specific thyroid cancers, including PTC and MTC [324]. Other meta-analyses reported null associations between GLP-1RA therapy and overall thyroid cancer risk, further complicating the interpretation of these findings. This inconsistency suggests that while the potential risk may exist, it is not yet definitively established [325].

Fears regarding the potential link between GLP-1RAs and pancreatic cancer have also garnered attention, primarily due to the known association between GLP-1RAs and pancreatitis, which is a risk factor for pancreatic malignancy [326]. However, accumulating clinical evidence has largely refuted a causal link between GLP-1RAs and pancreatic cancer [327,328], with some studies even suggesting a potential protective effect [329]. A notable large-scale population-based cohort study with a seven-year follow-up demonstrated no increased incidence of pancreatic cancer among GLP-1RA users. However, the study authors recommended continued surveillance beyond the follow-up period due to the long latency period of pancreatic cancer [330].

Beyond thyroid and pancreatic cancers, research examined the broader impact of GLP-1RAs on various other malignancies. Emerging evidence suggests that GLP-1RAs may confer protective effects against certain cancers when compared to other anti-diabetic therapies. For instance, GLP-1RAs were associated with a reduced risk of HCC and hepatic decompensation in adults with T2DM [331]. Similarly, a nationwide Danish cohort study reported a lower incidence of prostate cancer in individuals using GLP-1RAs compared to those on basal insulin, particularly in older adults and those with preexisting CV disease [332]. Further supporting these potential anti-cancer benefits, a large, real-world database analysis of 112,000 patients treated with GLP-1RAs found reductions in the risks of lung, colon, bladder, and prostate cancers [333]. A comprehensive retrospective cohort study utilizing a nationwide electronic health records database of over 1.6 million diabetic patients revealed that GLP-1RAs were associated with significantly reduced risks for 10 out of 13 organ-associated cancers, when compared to insulin. Notable reductions were observed in gallbladder cancer, meningioma, and pancreatic cancer. However, the study did not find significant associations between GLP-1RAs and breast or thyroid cancers, while an increased risk of kidney cancer was observed when compared to metformin [334].

#### 5.4.3. Tirzepatide

Current evidence on the role of tirzepatide in cancer development remains limited and inconclusive. A recent meta-analysis of phases 2 and 3 randomized RCTs investigating tirzepatide in T2DM patients found no significant increase in cancer risk associated with its use. This meta-analysis, which included nine RCTs, did not identify a statistically significant elevation in the incidence of any type of cancer, either as a primary or secondary outcome. However, it is important to consider the study’s limitations, including small sample sizes, relatively short durations (ranging from 36 to 72 weeks), and the limited number of reported cancer events. These constraints necessitate cautious interpretation of the findings, as the relatively short follow-up periods may not capture the full latency period for many cancers [335]. Moreover, data from the U.S. Food and Drug Administration Adverse Event Reporting System (FAERS) sparked issues about a potential association between tirzepatide and an increased reporting of MTC. Specifically, there was a 13.67-fold higher likelihood of reported MTC cases in patients treated with tirzepatide compared to other medications. While this signal warrants attention, it is important to note that adverse event reporting databases are subject to various biases and confounders and do not establish causality. The overall safety profile of tirzepatide appears to be similar to that of other GLP-1RAs, indicating that, apart from the potential increased risk of MTC, tirzepatide’s cancer risk seems aligned with established therapies in its class [336].

### 5.5. SGLT-2 Inhibitors

In contrast to the well-documented cardioprotective and nephroprotective effects of the SGLT-2 inhibitors, worries emerged about potential cancer risks, specifically regarding breast and bladder cancers. Early preclinical studies involving dapagliflozin raised fears due to observed, though statistically non-significant, increases in breast cancer incidence in females and bladder cancer incidence in males. These considerations were significant enough to delay dapagliflozin’s approval by the U.S. FDA in 2012, underscoring the need for further investigation into whether these risks translate to clinical outcomes [337]. Recent evidence generally alleviated questions regarding breast cancer risk. A large-scale study following 2154 breast cancer cases for an average of 2.2 years found no significant association between SGLT-2 inhibitors and breast cancer risk, irrespective of patient age or medication duration. The study included comparisons to DPP-4 inhibitors, with similar neutral findings regarding breast cancer [338]. Furthermore, a population-based cohort analysis of 60,112 T2DM patients revealed a significant reduction in breast cancer risk associated with dapagliflozin, suggesting that early issues may have been due to detection bias rather than an actual increase in incidence [339]. Despite this growing body of evidence suggesting a neutral or even protective effect, long-term data remain crucial to fully confirm the absence of risk [337,340].

Bladder cancer, particularly in relation to empagliflozin, is also a subject of ongoing debate. The EMPA-REG OUTCOME (Empagliflozin Cardiovascular Outcome Event Trial in Type 2 Diabetes Mellitus Patients–Removing Excess Glucose) clinical trial reported a small number of bladder cancer cases in empagliflozin users, with none reported in the placebo or comparator groups [341,342]. The low absolute number of events complicates interpretation, as bladder cancer typically progresses subtly and over an extended period, often spanning many years. Moreover, the increased glucose excretion caused by SGLT-2 inhibitors could potentially result in more frequent urinary tract infections and genital issues, leading to earlier cancer detection [340]. A meta-analysis encompassing 76 trials with over 116,000 participants similarly concluded that SGLT-2 inhibition is unlikely to significantly impact the risk of breast or bladder cancers, adding to the growing consensus that initial safety concerns may have been overstated [343].

Beyond breast and bladder cancers, emerging evidence points to potential protective effects of SGLT-2 inhibitors against other malignancies. A Mendelian randomization study suggested a lower risk of prostate cancer associated with SGLT-2 inhibition, potentially related to reductions in prostate-specific antigen (PSA) levels and uridine metabolism [344]. Similarly, canagliflozin demonstrated a protective effect against gastrointestinal cancers, although these findings require further validation in larger clinical trials [345]. Interestingly, a recent meta-analysis of 58 studies involving over 113,000 participants found no significant increase in overall cancer risk with these agents, though specific drug differences were noted. Ertugliflozin was associated with a higher overall incidence of cancer, while dapagliflozin demonstrated a 47% reduction in bladder cancer risk and a 26% reduction in respiratory cancer risk. However, dapagliflozin and ertugliflozin were linked to a potentially elevated risk of kidney cancer compared to placebo [346].

In conclusion, the impact of anti-diabetic drugs on cancer risk and outcomes presents a complex landscape requiring careful consideration. Metformin showed promise in lowering cancer risk and enhancing survival across various cancers. Despite these encouraging data, it has yet to receive formal approval for this purpose. This lack of endorsement largely stems from inconsistent study findings, which may be influenced by factors such as dosage, treatment duration, and individual patient characteristics. Interpreting these results warrants thoughtful analysis, as observational studies and meta-analyses often display high variability and are susceptible to biases, including immortal time bias, which can distort outcomes. Notably, a large phase 3 randomized trial involving 3649 women treated over five years found no significant benefit from metformin as an adjunct therapy for breast cancer in terms of disease-free or overall survival [347].

Evidence suggests hyperinsulinemia as a potential risk factor for cancer development, yet findings remain conflicting. This uncertainty may stem from the distinction between the controlled therapeutic use of exogenous insulin and the endogenous overproduction observed in insulin resistance. Exogenous insulin therapy aims to maintain euglycemia, thereby potentially mitigating the harmful effects of hyperglycemia, such as inflammation, immune dysfunction, and tumor promotion. Additionally, treatments like oral insulin secretagogues (e.g., SUs) showed favorable impacts on certain cancers, highlighting the complex and context-dependent role of insulin in cancer dynamics. Furthermore, the association between pioglitazone and cancer remains contentious, with bladder cancer being the most reported concern. Recent studies raised questions about this association, yet caution is still advised, especially for patients with a history of hematuria or bladder cancer.

Incretin-based therapies present a dynamic area of research, showing both therapeutic benefits and ongoing safety concerns. Although large-scale studies like LEADER (Liraglutide Effect and Action in Diabetes) have alleviated initial worries regarding the risk of thyroid malignancies, concerns remain, particularly for individuals with a family history of MEN2 [348]. Newer agents, such as tirzepatide, appear to pose no overall increased cancer risk, yet continued monitoring is vital, particularly regarding associations with MTC. Finally, early concerns regarding SGLT-2 inhibitors and increased risks of breast and bladder cancers have largely been dispelled. On the other hand, drug-specific effects, notably dapagliflozin’s protective benefits against bladder cancer and ertugliflozin’s potential link to renal cancer, highlight the importance of individualized risk assessments.

## 6. Conclusions

In recent years, anti-diabetic medications have garnered significant attention for their potential impact on cancer biology and therapeutic outcomes. Among these treatments, metformin stands out as the most extensively studied, with substantial evidence supporting its role in inhibiting tumor cell viability across various cancer types. In contrast, the efficacy of other glucose-lowering conventional therapies, such as insulin, oral insulin secretagogues, pioglitazone, and DPP-4 inhibitors, remains controversial or, in some cases, unfavorable. Furthermore, early findings suggest that novel therapies, particularly SGLT-2 inhibitors, may offer notable anti-cancer benefits, likely due to their broad pleiotropic effects. Despite these promising developments, conclusive evidence regarding the role of anti-diabetic therapies in cancer prevention and treatment remains elusive. To date, no anti-diabetic drug has been formally approved for anti-cancer use. Furthermore, even in cases like pioglitazone and GLP-1 analogues, where their use is discouraged in specific malignancies, some studies present conflicting results. The divergent findings observed were likely influenced by discrepancies between animal models and human trials, as well as variations in clinical trial designs, dosing regimens, and patient demographics. These limitations highlight the need for caution but do not diminish the potential significance of anti-diabetic treatments in tumorigenesis. To move this field forward, future studies must address these challenges through robust methodologies, large and diverse participant cohorts, and extended follow-up periods. Such efforts are essential to fully understand the oncological implications of anti-diabetic therapies, ensuring that their integration into cancer care is both evidence based and tailored to patient safety.

## Figures and Tables

**Table 1:** The impact of anti-diabetic drugs on cancer development and progression across different cancer types.

Cancer Type	Anti-Diabetic Drugs and Their Impact on Cancer-Related Signaling
Brain cancer	- Metformin targets GB-initiating cells through the AMPK-FOXO3 axis [141] - Insulin facilitates GB proliferation and survival by activating the Akt pathway [49] - Glibenclamide inhibits GB growth by promoting intracellular acidification through downregulation of Kir4.1 and MCT1 expression at specific doses [142] - Pioglitazone’s impact on GB is heterogeneous; significantly dampens cell viability and proliferation in only a subset, without increasing differentiation or affecting the Wnt/β-catenin pathway to a notable extent [143] - Linagliptin exerts anti-tumor effects on GB cells potentially through modulation of proteins involved in the cell cycle and adhesion via phosphorylated NF-kB regulation [144] - Exenatide attenuates GB cell migration and invasion through the GLP-1R/SIRT3 signaling pathway [106] - Canagliflozin reduces GB cell proliferation and glucose uptake by favoring AMPK phosphorylation and suppressing p70 S6 kinase and S6 ribosomal protein activity [123]
Thyroid cancer	- At concentrations of 0.1 mM and above, metformin increases the percentage of apoptotic cells and promotes G0/G1 phase cell cycle arrest, with no effect on the DNA repair response at concentrations as low as 0.3 mM [145] - Both human insulin and insulin glargine facilitate thyroid cell proliferation at high doses, enhancing the phosphorylation of IR, Akt, and ERK1/2 in a dose-dependent manner, with glargine showing longer-lasting effects; however, therapeutic doses do not stimulate cell proliferation [50] - PPAR-γ agonistic effect of pioglitazone prevents metastatic thyroid cancer by promoting adipocyte-like trans-differentiation of thyroid carcinoma cells [146] - Saxagliptin boosts migration and invasion of PTC through the Nrf2/HO-1 signaling pathway [89] - Liraglutide may mitigate cell growth and migration in both PTC and MTC by modulating the PI3K/Akt/mTOR pathway in a dose- and time-dependent manner [101] - Canagliflozin reduces cell viability and colony formation while lowering pro-tumor chemokines, such as CXCL8 and CCL2, which are involved in enhanced cell migration and endothelial proliferation [124]
Breast cancer	- Metformin promotes survival of dormant ER^+^ by upregulating AMPK [25] - Insulin can regulate breast cancer growth by activating both insulin and IGF receptors [147] - Glipizide blocks angiogenesis by modulating VEGF/VEGFR2 signaling [67] - Pioglitazone mitigates cancer cell proliferation and migration by modulating the JAK/STAT3 signaling pathway [75] - DPP-4i favors EMT and metastatic potential through the CXCL12/CXCR4 signaling pathway [87] - At high concentrations, liraglutide may promote cancer progression via the NOX4/ROS/VEGF signaling pathway [109] - Ipragliflozin attenuates cell proliferation by modifying membrane dynamics and simulating glucose deprivation effects [129]
Lung cancer	- Metformin reduces oncogenic markers like HES1 and REDD1, while modulating p-mTOR and p53 levels, resulting in diminished tumor cell proliferation [37] - Insulin favors the proliferation and migration of cancer cells by upregulating the PI3K/Akt pathway [51] - Glibenclamide targets SUR1 to inhibit cell growth, migration, and EMT by reducing p70S6K activity and increasing the tumor suppressor KLF4 [148] - In combination with celecoxib, pioglitazone decreases tumor weight and increases survival by mitigating NF-κB-mediated proliferation [76] - Vildagliptin inhibits lung metastases by hindering autophagy, promoting apoptosis, and modulating the cell cycle [149] - Liraglutide hinders lung cancer cell proliferation, migration, and EMT, while also demonstrating anti-aging effects by debilitating cellular senescence and ER stress] [150] - Canagliflozin disrupts HIF-1α stabilization, impairing mitochondrial function and survival pathways by inhibiting mTOR and HDAC2 [133]
Hepatocellular carcinoma	- Metformin activates AMPK to disrupt lipid metabolism, enhances apoptosis and oxidative stress via p38MAPK activation [41] - Insulin promotes cell proliferation and survival by activating PI3K/Akt and Ras/MAPK pathways [52] - Glibenclamide inhibits Kv channels, resulting in a dose- and calcium-dependent decrease in the adhesion and proliferation of tumor cells [63] - Pioglitazone shows anti-fibrotic and hepatoprotective effects, potentially by modulating the MAPK and AMPK signaling pathways [79] - Linagliptin inhibits tumor cell growth by modulating ADORA3, inducing apoptosis and increasing cAMP levels [151] - Liraglutide may enhance anti-tumor immune responses through the IL-6/STAT3 signaling pathway [117] - Canagliflozin may mitigate liver steatosis, fibrosis, and tumor development, while promoting healthier adipose tissue with lower oxidative stress [130]
Pancreatic cancer	- Metformin facilitates apoptosis in cancer cells by modulating histone acetyltransferases (PCAF, p300, CBP) and SIRT1 expression [152] - Insulin induces the growth and fibrosing responses of PaSC through activation of the IR/IGF-1R, which in turn enhance Akt/mTOR/p70S6K signaling and reduce FOXO1, contributing to cell proliferation and extracellular matrix production [153] - Gliclazide favors DNA repair in cancer cells by stimulating NER and non-NHEJ pathways, without affecting these processes in normal human cells [154] - Pioglitazone blocks metastasis by altering inflammation-related gene expression, including CEA and COX-2 [80] - Saxagliptin promotes β-cell proliferation by elevating stromal cell-derived factor-1α [90] - Liraglutide inhibits cancer growth and promotes apoptosis by downregulating the PI3K/Akt and ERK1/2 signaling pathways [103] - Canagliflozin exhibits anti-tumor activity in cancer cells by downregulating the PI3K/Akt/mTOR pathway and impairing glycolysis [136]
Colorectal cancer	- Metformin mitigates cancer cell proliferation by targeting INHBA, inhibiting TGF-β/PI3K/Akt signaling and causing G1/S cell cycle arrest [155] - Insulin promotes cancer progression and metastasis by upregulating ACAT1 [53] - Glibenclamide and miR-223, by inhibiting NLRP3 in cancer cells, mitigate cell growth and migration, but miR-223 has a more pronounced effect on apoptosis and cytokine secretion, though neither fully prevents metastasis [156] - Pioglitazone mitigates cancer stem cell viability and increases MET, aiding in cancer suppression [82] - Sitagliptin attenuates cancer metastasis by reducing cell invasion, motility, and EMT through DPP4-dependent mechanisms [94] - GLP-1 RAs diminish cell cycle progression and promote apoptosis through the PI3K/Akt/mTOR pathway [119] - Canagliflozin shows potential efficacy by disrupting cellular metabolism and inducing ER stress, which in turn promotes autophagy and apoptosis via SIRT3 upregulation [135]
Bladder cancer	- Metformin suppresses the migration and proliferation of cancer cells and promotes apoptosis, likely by inhibiting the PI3K/Akt/mTOR pathway [157] - Both high-dose human insulin and insulin glargine promote tumor cell proliferation by activating Akt phosphorylation through a PI3K-independent pathway [158] - Pioglitazone causes DNA damage and promotes malignant transformation by altering gene expression and inducing EMT [69] - DDP-4i may reduce tumor aggressiveness by impairing cancer cell viability, proliferation, migration, and invasion [159] - Empagliflozin induces dysplastic changes in urothelium, with decreased expression of CK-7 and CK-8 and increased expression of CK-20 and Ki-67, suggesting heightened proliferative activity [160]
Prostate cancer	- Metformin induces oxidative stress in cancer cells by promoting ROS and disrupting oxidative phosphorylation, leading to redox imbalance and enhanced apoptosis [40] - Glipizide inhibits angiogenesis via the HMGIY/Angiopoietin-1 signaling pathway, without affecting cancer cell proliferation [161] - Pioglitazone alleviates inflammation in periprostatic WAT, potentially impacting cancer progression by modulating adipose tissue-related inflammatory responses [73] - Exenatide may lower tumor cancer progression through the inhibition of ERK-MAPK signaling [162] - Canagliflozin inhibits tumor cell growth by mitigating mitochondrial respiration and ATP production. This mechanism involves activation of AMPK, reduced lipid synthesis, and decreased glucose uptake [138].
Ovarian cancer	- Metformin alleviates cell proliferation by activating the AMPK/GSK3β pathway, resulting in cyclin D1 degradation and G1 phase cell cycle arrest [34] - Through IGF-I, insulin induces COX-2 expression, which enhances VEGF production and PGE2 biosynthesis. This process activates PI3K, MAPK, and PKC pathways and promotes SKOV-3 cell migration by favoring uPA over PAI-1 via the PI3K/AKT pathway [163]. - Glibenclamide dampens angiogenesis and metastasis in ovarian cancer by reducing cellular invasion and migration through suppression of PDGF-AA secretion [65] - Ciglitazone and troglitazone reduce ovarian cancer cell proliferation, while pioglitazone has no effect, suggesting that the observed impact is PPAR-γ independent [164] - Sitagliptin promotes apoptosis via caspase 3/7 activation and suppresses migration and invasiveness in SKOV-3 cells, while also reducing the production of MMPs and TIMPs [165] - Exenatide may lower ovarian cancer aggressiveness by debilitating cell migration, inducing apoptosis, and modulating metalloproteinase expression [114]
Endometrial cancer	- Metformin activates AMPK, leading to enhanced TET2 gene expression and suppression of cancer cell growth [32] - Insulin stimulates aromatase activity in both endometrial glands and stroma, suggesting that hyperinsulinemia may increase the risk of estrogen-dependent endometrial neoplasia by enhancing local estrogen production [166] - Pioglitazone demonstrates significant dose-dependent anti-cancer activity by improving body weight, survival time, and uterine tissue weight [167] - DPP-4 overexpression accelerates carcinoma progression by enhancing cell proliferation, invasion, and HIF-1a-VEGFA signaling, while pharmacological inhibition with sitagliptin shows potential as an effective therapeutic strategy [92] - Tirzepatide mitigates tumor growth by altering glycolysis and ErbB signaling in obese mice, while enhancing glycosylation and phospholipase D signaling in lean mice [113]
Other malignancies	- Metformin inhibits the invasion and proliferation of cervical cancer cells by regulating the insulin signaling pathway and upregulating the expression of the tumor suppressor IGFBP7 [168] - Metformin mitigates MPM cell proliferation and promotes apoptosis by decreasing Notch1 activation [169] - Sitagliptin affects gastric cancer cell proliferation by reducing MAGE-A3 expression through the inactivation of YAP [170] - Dapagliflozin exerts cytotoxic effects in RCC by decreasing glucose uptake, disrupting cell cycle progression, and promoting apoptosis [137]

Abbreviations: ACAT1: acetyl-CoA acetyltransferase 1; ADORA3: adenosine A3 receptor; Akt: protein kinase B; AMPK: AMP-activated protein kinase; ATP: adenosine triphosphate; cAMP: cyclic adenosine monophosphate; CBP: CREB-binding protein; CCL2: C-C motif chemokine ligand 2; COX-2: cyclooxygenase-2; CXCR4: C-X-C chemokine receptor type 4; CXCL8: C-X-C motif chemokine ligand 8; CXCL12: C-X-C motif chemokine ligand 12; DPP-4: dipeptidyl peptidase 4; DDP-4i: dipeptidyl peptidase-4 inhibitor; EMT: epithelial-to-mesenchymal transition; ER: endoplasmic reticulum; ERK1/2: extracellular signal-regulated kinases 1 and 2; FOXO1: Forkhead box O1; FOXO3: Forkhead box O3; GB: glioblastoma; GLP-1 RAs: glucagon-like peptide-1 receptor agonists; GLP-1R: glucagon-like peptide-1 receptor; GSK3β: glycogen synthase kinase 3 beta; HES1: hairy and enhancer of split-1; HGF: hepatocyte growth factor; HIF-1α: hypoxia-inducible factor 1-alpha; HMGIY: high-mobility group protein I(Y); IGF: insulin-like growth factor; IGF-I: insulin-like growth factor I; IGF-1R: insulin-like growth factor 1 receptor; IGFBP7: insulin-like growth factor-binding protein 7; IL-6: interleukin-6; INHBA: inhibin subunit beta A; IR: insulin receptor; JAK: Janus kinase; Ki-67: antigen Kiel 67; KLF4: Kruppel-like factor 4; MAGE-A3: melanoma-associated antigen 3; MAPK: mitogen-activated protein kinase; MCT1: monocarboxylate transporter 1; MET: mesenchymal-to-epithelial transition; MMPs: matrix metalloproteinases; MMP-2: matrix metalloproteinase-2; mM: millimolar; mTOR: mechanistic target of rapamycin; NER: nucleotide excision repair; NHEJ: non-homologous end joining; NF-κB: nuclear factor kappa-light-chain-enhancer of activated B cells; NLRP3: NLR family pyrin domain containing 3; Notch1: neurogenic locus notch homolog protein 1; NOX4: NADPH oxidase 4; p38MAPK: p38 mitogen-activated protein kinase; p70 S6 kinase: 70 kDa ribosomal protein S6 kinase; PAI-1: plasminogen activator inhibitor-1; PaSC: pancreatic stellate cell; PGE2: prostaglandin E2; PI3K: phosphoinositide 3-kinase; PKC: protein kinase C; p-mTOR: phosphorylated mechanistic target of rapamycin; PPAR-γ: peroxisome proliferator-activated receptor gamma; PTC: papillary thyroid carcinoma; Ras: rat sarcoma; RCC: renal cell carcinoma; REDD1: regulated in development and DNA damage responses 1; ROS: reactive oxygen species; SIRT: sirtuin; SKOV-3: a human ovarian cancer cell line; STAT3: signal transducer and activator of transcription 3; TET2: Ten-Eleven Translocation methylcytosine dioxygenase 2; TGF-β: transforming growth factor beta; TIMP: tissue inhibitor of metalloproteinases; VEGFA: vascular endothelial growth factor A; VEGF: vascular endothelial growth factor; VEGFR2: vascular endothelial growth factor receptor 2; WAT: white adipose tissue; Wnt: wingless-type MMTV integration site family; YAP: yes-associated protein.

## Data Availability

Not applicable.

## Abbreviations

4E-BP1: Eukaryotic translation initiation factor 4E-binding protein 1; 5-FU: 5-fluorouracil; ACAT1: Acetyl-CoA acetyltransferase 1; ADORA3: Adenosine A3 receptor; AKI: Acute kidney injury; Akt: Protein kinase B; AMPK: AMP-activated protein kinase; ATP: Adenosine triphosphate; Bcl-2: B cell lymphoma 2; BMI: Body mass index; BUBR1: BUB1 mitotic checkpoint kinase 1; CAFs: Cancer-associated fibroblasts; cAMP: Cyclic adenosine monophosphate; CANA: Canagliflozin; CBP: CREB-binding protein; CCL2: C-C motif chemokine ligand 2; CD4^+^ T cells: Cluster of differentiation 4 positive T cells; CD8^+^ T cells: Cluster of differentiation 8 positive T cells; CKD: Chronic kidney disease; CML: Chronic myeloid leukemia; COX-2: Cyclooxygenase-2; CRC: Colorectal carcinoma; CV: Cardiovascular; CXCL: C-X-C motif chemokine ligand 12; CXCL8: C-X-C motif chemokine ligand 8; CXCL12: C-X-C motif chemokine ligand 12; CXCR: C-X-C chemokine receptor; CXCR4: C-X-C chemokine receptor type 4; DAPA: Dapagliflozin; DC: Dendritic cell; DLBCL: Diffuse large B cell lymphoma; DM: Diabetes mellitus; EMT: Epithelial-to-mesenchymal transition; ER: Endoplasmic reticulum; ER^+^: Estrogen receptor-positive; ERK: Extracellular signal-regulated kinase; DOX: Doxorubicin; DPP-4: Dipeptidyl peptidase-4; DPP-4i: Dipeptidyl peptidase-4 inhibitor; dsDNA: Double-stranded DNA; eGFR: Estimated glomerular filtration rate; EMPA: Empagliflozin; EMPA-REG OUTCOME: The Empagliflozin Cardiovascular Outcome Event Trial in Type 2 Diabetes Mellitus Patients–Removing Excess Glucose; FAERS: U.S. Food and Drug Administration Adverse Event Reporting System; FDA: Food and Drug Administration; FOXO1: Forkhead box O1; FOXO3: Forkhead box O3; FXR: Farnesoid X receptor; GB: Glioblastoma; GIP: Glucose-dependent insulinotropic polypeptide; GLP-1 RAs: Glucagon-like peptide-1 receptor agonists; GLP-1R: Glucagon-like peptide-1 receptor; GM-CSF: Granulocyte-macrophage colony-stimulating factor; GPx: Glutathione peroxidase; GSK3β: Glycogen synthase kinase 3 beta; HCC: Hepatocellular carcinoma; HDAC2: Histone deacetylase 2; HES1: Hairy and enhancer of split-1; HGF: Hepatocyte growth factor; HIF-1α: Hypoxia-inducible factor 1-alpha; HMGB1: High-mobility group box 1; HMGIY: High-mobility group protein I(Y); HO-1: Heme oxygenase-1; HSF1: Heat shock factor 1; ICAM-1: Intercellular adhesion molecule-1; ICI: Immune checkpoint inhibitor; IFN-β: Interferon-beta; IFN-γ: Interferon-gamma; IGF: Insulin-like growth factor; IGF-1: Insulin-like growth factor 1; IGF-1R: Insulin-like growth factor receptor; IGFBP7: Insulin-like growth factor-binding protein 7; IL: Interleukin; INHBA: inhibin subunit beta A; IR: Insulin receptor; IRF3: Interferon regulatory factor 3; IRS-1: Insulin receptor substrate-1; JAK: Janus kinase; JNK: c-Jun N-terminal kinase; Ki-67: Antigen Kiel 67; KLF4: Kruppel-like factor 4; Kv: Voltage-gated potassium; LDH: Lactate dehydrogenase; LEADER: Liraglutide Effect and Action in Diabetes trial; LINA: Linagliptin; LIRA: Liraglutide; LVEF: Left ventricular ejection fraction; MACE: Major adverse cardiovascular events; MAFLD: Metabolic dysfunction-associated fatty liver disease; MAGE-A3: Melanoma-associated antigen 3; MAPK: Mitogen-activated protein kinase; MCT1: Monocarboxylate transporter 1; MDA: Malondialdehyde; MDSCs: Myeloid-derived suppressor cells; MET: Mesenchymal-to-epithelial transition; miR: MicroRNA; mM: millimolar; MMP: Matrix met-alloproteinase; MTC: Medullary thyroid cancer; mRNA: Messenger RNA; MTF: Metformin; mTOR: Mammalian target of rapamycin; mTORC1: Mammalian target of rapamycin complex 1; MyD88: Myeloid differentiation factor 88; NER: Nucleotide excision repair; NETs: Neutrophil ex-tracellular traps; NF-κB: Nuclear factor kappa-light-chain-enhancer of activated B cells; NHEJ: Non-homologous end joining; NK: Natural killer; NLRP3: NLR family pyrin domain containing 3; Notch1: Neurogenic locus notch homolog protein 1; NOX4: NADPH oxidase 4; Nrf2: Nuclear factor erythroid 2-related factor 2; NSCLC: Non-small cell lung cancer; OPA-1: Protein optic atrophy-1; OS: Overall survival; OTUD5: OTU deubiquitinase 5; OXPHOS: Oxidative phosphorylation; p-AMPK: Phosphorylated AMPK;p38MAPK: p38 mitogen-activated protein kinase; p70 S6 kinase: 70 kDa ribosomal protein S6 kinase; PAI-1: Plasminogen activator inhibitor-1; PanIN: Pancreatic intraepithelial neoplasia; PARP: Cleaved-poly-ADP ribose polymerase; PaSC: Pancreatic stellate cell; PD-1: Programmed cell death protein-1; PDGF-AA: Platelet-derived growth factor-AA; PD-L1: Programmed death-ligand-1; PGC-1α: Peroxisome proliferator-activated receptor gamma coactivator-1 alpha; PGE2: Prostaglandin E2; PFS: Progression-free survival; PI3K: Phosphoinositide 3-kinase; PIO: Pioglitazone; PKC: Protein kinase C; PKM2: Pyruvate kinase M2; p-mTOR: Phosphorylated mechanistic target of rapamycin; PPAR-γ: Peroxisome proliferator-activated receptor gamma; PSA: Prostate-specific antigen; PTC: Papillary thyroid carcinoma; Raptor: Regulatory-associated protein of mTOR; Ras: Rat sarcoma; RCC: Renal cell carcinoma; REDD1: Regulated in development and DNA damage response 1; ROS: Reactive oxygen species; SAC: Spindle assembly checkpoint; SAXA: Saxagliptin; SCFAs: Short-chain fatty acids; SGLT-2: Sodium-glucose co-transporter-2; SIRT: sirtuin; SKOV-3: a human ovarian cancer cell line; STAT3: Signal transducer and activator of transcription 3; STING: Stimulator of interferon genes; SUs: Sulfonylureas; SUR1: Sulfonylurea receptor 1; T1DM: Type 1 diabetes mellitus; T2DM: Type 2 diabetes mellitus; TAMs: Tumor-associated macrophages; TET2: Ten-Eleven Translocation methyl-cytosine dioxygenase 2; TGF-β: Transforming growth factor-beta; TIL: Tumor-infiltrating lympho-cyte; TIMP: Tissue inhibitor of metalloproteinases; TLR4: Toll-like receptor 4; TME: Tumor microen-vironment; TNBC: Triple-negative breast cancer; TNF: Tumor necrosis factor; Tregs: Regulatory T cells; TSC2: Tuberous sclerosis complex 2; ULK1: Unc-51-like autophagy activating kinase 1; VCAM-1: Vascular cell adhesion molecule-1; VEGF: Vascular endothelial growth factor; VEGFA: Vascular endothelial growth factor A; VEGFR2: Vascular endothelial growth factor receptor 2; WAT: White adipose tissue; WHO: World Health Organization; Wnt: Wingless-type MMTV integration site family; YAP1: Yes-associated protein 1; ZFP36: Zinc finger protein 36.
